# HIV-1 Vpr drives a tissue residency-like phenotype during selective infection of resting memory T cells

**DOI:** 10.1016/j.celrep.2022.110650

**Published:** 2022-04-13

**Authors:** Ann-Kathrin Reuschl, Dejan Mesner, Maitreyi Shivkumar, Matthew V.X. Whelan, Laura J. Pallett, José Afonso Guerra-Assunção, Rajhmun Madansein, Kaylesh J. Dullabh, Alex Sigal, John P. Thornhill, Carolina Herrera, Sarah Fidler, Mahdad Noursadeghi, Mala K. Maini, Clare Jolly

**Affiliations:** 1 Division of Infection and Immunity, University College London, London WC1E 6BT, UK; 2 Department of Cardiothoracic Surgery, University of KwaZulu-Natal, Durban 4091, South Africa; 3 Centre for the AIDS Programme of Research in South Africa, Durban 4091, South Africa; 4 Africa Health Research Institute, Durban 4001, South Africa; 5 School of Laboratory Medicine and Medical Sciences, University of KwaZulu-Natal, Durban 4091, South Africa; 6 Max Planck Institute for Infection Biology, 10117 Berlin, Germany; 7 Peter Medawar Building for Pathogen Research, Nuffield Department of Medicine, University of Oxford, Oxford OX1 3XY, UK; 8 Department of Infectious Disease, Faculty of Medicine, Imperial College, London W2 1NY, UK; 9 Imperial College NIHR Biomedical Research Centre, London W2 1NY, UK

**Keywords:** HIV-1, Vpr, resting memory T cell, cell-cell, tissue residency, permissivity, transcriptional reprogramming

## Abstract

HIV-1 replicates in CD4^+^ T cells, leading to AIDS. Determining how HIV-1 shapes its niche to create a permissive environment is central to informing efforts to limit pathogenesis, disturb reservoirs, and achieve a cure. A key roadblock in understanding HIV-T cell interactions is the requirement to activate T cells *in vitro* to make them permissive to infection. This dramatically alters T cell biology and virus-host interactions. Here we show that HIV-1 cell-to-cell spread permits efficient, productive infection of resting memory T cells without prior activation. Strikingly, we find that HIV-1 infection primes resting T cells to gain characteristics of tissue-resident memory T cells (T_RM_), including upregulating key surface markers and the transcription factor Blimp-1 and inducing a transcriptional program overlapping the core T_RM_ transcriptional signature. This reprogramming is driven by Vpr and requires Vpr packaging into virions and manipulation of STAT5. Thus, HIV-1 reprograms resting T cells, with implications for viral replication and persistence.

## Highlights

• Cell-to-cell spread makes resting CD4^+^ T cells permissive to HIV-1 infection
• Resting memory T cells are productively infected by HIV-1
• HIV-1 Vpr drives resting memory T cells to gain characteristics of tissue residency
• Vpr induces widespread transcriptional reprogramming of infected T cells

## Graphical abstract

**Figure:**
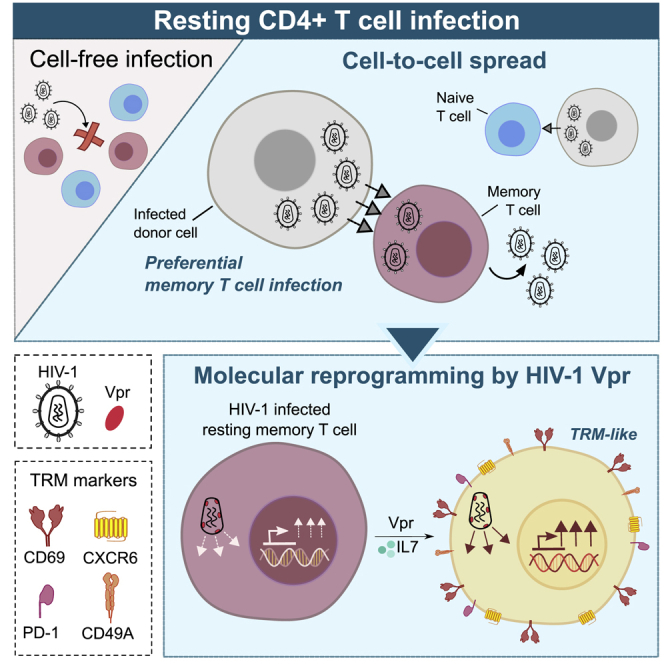

## Teaser

Reuschl et al. show that HIV-1 cell-to-cell spread promotes productive infection of resting T cells, bypassing the need for *in vitro* activation. They discover that HIV-1 Vpr reprograms resting T cells phenotypically and transcriptionally to gain characteristics of tissue-resident memory T cells, suggesting that HIV-1 drives changes that may affect viral reservoir establishment and persistence.

## Introduction

Resting primary CD4^+^ T cells cannot be efficiently infected by cell-free HIV-1 virions *in vitro* and require robust mitogenic stimulation to support viral replication (Stevenson et al., 1990; Swiggard et al., 2005; Zack et al., 1990). This has led to the notion that T cell activation is necessary for HIV-1 replication. However, mitogenic T cell activation *in vitro* results in widespread phenotypic and functional reprogramming, which dominates changes in gene and protein expression (Howden et al., 2019; Szabo et al., 2019a; Wolf et al., 2020), concealing and potentially altering authentic virus-host interactions. This presents a significant challenge for understanding the cellular response to HIV-1 infection and the consequences of the virus-host interaction for HIV-1 replication and persistence. While it is clear that HIV-1 efficiently infects and replicates in activated T cells, the outcomes of the virus-host interaction with resting T cells have been reported to be cell death (Doitsh et al., 2010) or latency (Agosto et al., 2018). However, previous data demonstrating that HIV-1 cell-to-cell spread is highly efficient and drives widespread changes in protein phosphorylation status in both infected and target cells (Hübner et al., 2009; Jolly et al., 2004; Len et al., 2017; Sattentau, 2008; Sourisseau et al., 2007) suggested that cell-to-cell spread may overcome the barrier to productive infection of resting T cells. Here, we comprehensively show for the first time that HIV-1 exploits cell-to-cell spread to efficiently infect resting memory CD4^+^ T cells, and we have used this to uncover a hitherto unknown consequence of HIV-1 infection for T cell reprogramming driven by the accessory protein Vpr, inducing cells to gain characteristics of tissue-resident memory T cells.

## Results

### HIV-1 cell-to-cell spread drives productive infection of resting memory CD4^+^ T cells

**Figure 1:**
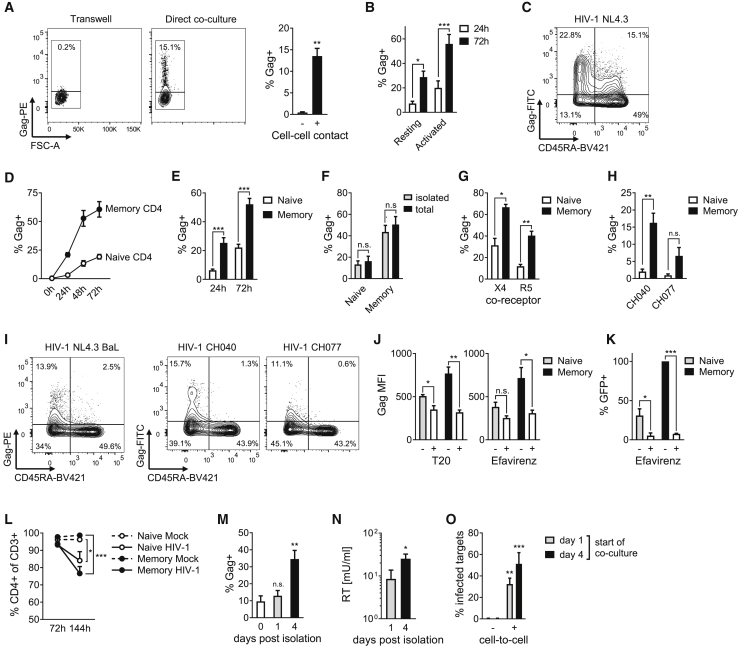
HIV-1 exploits cell-to-cell spread to preferentially infect resting memory CD4^+^ T cells (A) HIV-1 NL4.3 infected mitogenically activated primary CD4^+^ donor T cells co-cultured with resting autologous primary CD4^+^ target T cells separated by a 0.4 μm transwell (cell-free) or in direct co-culture (cell-cell). Target cell infection was measured by intracellular staining for HIV-1 Gag protein. Representative flow cytometry plots are shown. Bar graphs show mean of independent experiments (n = 4). (B) Cell-to-cell spread into resting or αCD3/αCD28-activated CD4^+^ target T cells measured by intracellular Gag expression (n = 5). (C and D) Cell-to-cell spread from activated primary donor CD4^+^ T cells to resting primary target CD4^+^ T cells preferentially infects CD45RA^-^ memory CD4^+^ T cells. A representative flow cytometry plot and quantification are shown (n = 4). (E) Quantification of infection performed as in (C) (n = 11). (F) HIV-1 infection of target CD4^+^ T cells as part of the total resting CD4^+^ T cell population (total) compared with pre-isolated naive and memory CD4^+^ target T cells (isolated) (n = 9). (G and H) Quantification of infection of CXCR4 (X4)- and CCR5 (R5)-tropic viruses (n = 4) (G) and transmitter/founder viruses HIV-1 CH040 and CH077 (n = 7) (H). (I) Representative flow cytometry plots of cell-to-cell infection of resting CD4^+^ T cells with CCR5-tropic HIV-1 NL4.3 BaL and transmitter founder viruses HIV-1 CH040 and CH077 as performed in (C). (J and K) Cell-to-cell infection of resting CD4^+^ T cells is reduced by the HIV-1 fusion inhibitor T20 (n = 6) (left) and the reverse transcriptase inhibitor efavirenz (n = 6) (right) measured by intracellular Gag staining (median fluorescence intensity, MFI) (J) or HIV-1 LTR-driven GFP-reporter gene expression (n = 4) (K). (L) HIV-1 infection downregulates CD4 expression. Shown is the percentage of CD4^+^ cells in the total CD3^+^ target cell population (n = 6). (M–O) Resting CD4^+^ memory T cells were isolated after 72 h of cell-to-cell spread by fluorescence-activated cell sorting (FACS) and cultured for 4 days. HIV-1 infection was measured by intracellular Gag staining (M) and virus release was measured by culture supernatant reverse transcriptase (RT) activity (N) (n = 5–7). T cells recovered at day 1 or 4 post-isolation were then cultured with uninfected eFluor450^+^ target Jurkat T cells, and infection of Jurkat T cells was measured after 72 h (O) (n = 3). All measurements were made after 72 h or at the indicated time post co-culture. Data are the mean ± SEM. Paired two-tailed t test or one-way ANOVA with Bonferroni post test was used. For (L), the median + IQR is shown and Friedman test with Dunn’s post test was used. For (O), unpaired one-tailed t test was used. ^∗^p < 0.05; ^∗∗^p < 0.01; ^∗∗∗^p < 0.001; n.s., not significant.

To test whether cell-to-cell spread allows for productive infection of resting T cells, HIV-1-infected primary CD4^+^ T cells were co-cultured with uninfected autologous resting CD4^+^ T cells (Figures 1A and S1A–S1E). We confirmed that CD4^+^ T cells isolated from peripheral blood display a resting phenotype by staining for Ki67, CD69, CD25, CD38, HLA-DR, and MCM2 (Figures S1C–S1E). Infection of resting target cells in the absence of mitogenic or cytokine activation was measured. Direct co-culture of infected and uninfected cells resulted in significant levels of HIV-1 infection of resting CD4^+^ target T cells (Gag^+^) measured by intracellular flow cytometry staining (Figures 1A and S1F). By contrast, resting CD4^+^ T cells were not infected (<1%) when cell-cell contact was prevented by separating the two cell populations by a transwell (Figures 1A and S1F), a condition that allows for only cell-free infection. As expected, mitogenic activation of primary target T cells made them more permissive to HIV-1 infection (Figures 1B and S1F–S1H), but as previously shown, infection was still substantially boosted by direct co-culture allowing for cell-to-cell spread (Figures S1F–S1H) (Hübner et al., 2009; Jolly et al., 2004; Sourisseau et al., 2007). Resting T cells remained refractory to cell-free infection even when incubated with high doses of virus (Figure S1I) (exceeding the concentration of virus detected in a cell-to-cell co-culture Figure S1J), while, as expected, activated CD4^+^ T cells could be infected by cell-free virus (Figure S1I). Thus, we show that resting T cells are highly refractory to cell-free HIV-1, but this can be overcome during infection mediated by cell-to-cell spread.

**Figure 2:**
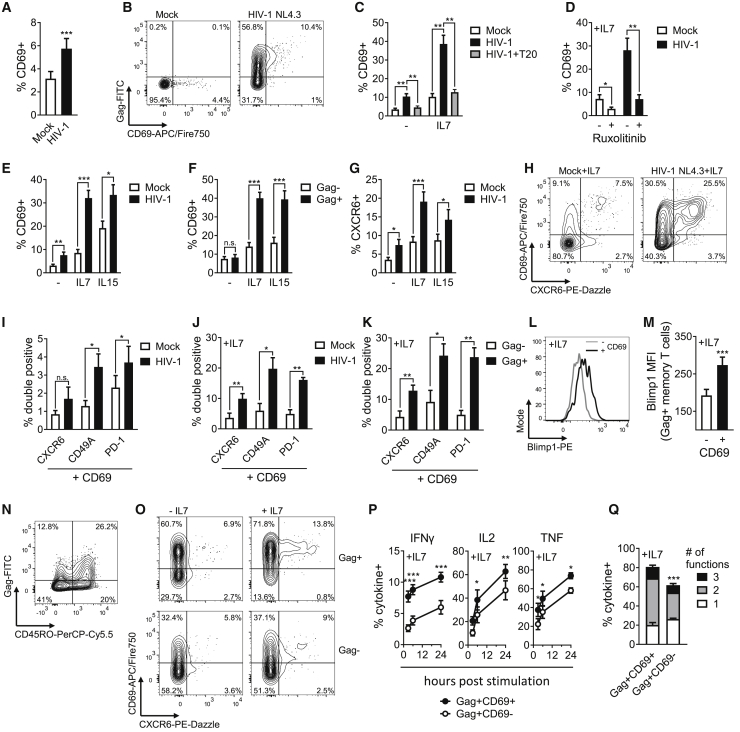
HIV-1 infection induces a T_RM_-like phenotype in resting memory CD4^+^ T cells (A) CD69 expression on resting memory CD4^+^ target T cells following co-culture with HIV-1-infected primary donor T cells or uninfected donor T cells (mock) (n = 17). (B) Representative flow cytometry plots from (A). (C) CD69 expression on infected resting memory CD4^+^ T cells ± IL-7 and T20 (n = 7). (D) CD69 expression on infected resting memory CD4^+^ T cells ± IL-7 and ruxolitinib (n = 8). (E) CD69 expression on infected resting memory CD4^+^ T cells in response to IL-7 and IL-15 (n = 11). (F) CD69 expression on infected Gag^+^ resting memory CD4^+^ T cells and uninfected Gag^−^ bystander cells in response to IL-7 and IL-15 (n = 11). (G) CXCR6 surface expression from (F) (n = 11). (H) Representative flow cytometry plots of CD69 and CXCR6 co-expression in the presence of IL-7. (I) Co-expression of CD69 with CXCR6, CD49A, or PD-1 on infected resting memory CD4^+^ T cells (n = 5–7). (J) As for (I) in the presence of IL-7 (n = 4–7). (K) As for (I) comparing infected Gag^+^ memory CD4^+^ T cells and uninfected Gag^−^ bystander cells. (L and M) Blimp-1 expression in CD69^+^ HIV-infected resting memory CD4^+^ T cells and infected CD69^−^ cells in the presence of IL-7 (n = 8). (N) Total lymphocytes from cellularized tonsils co-cultured with HIV-1-infected Jurkat T cells. Infection of resting CD4^+^ T cells shown as CD45RO versus Gag. (O) Representative flow cytometry plots of CD69 and CXCR6 co-expression on infected Gag^+^ and uninfected Gag^−^ tonsil resting memory CD4^+^ T cells ± IL-7. (P) Recall cytokine response by HIV-1-infected Gag^+^ resting memory T cells. At 72 h of co-culture, expression of IFN-γ, IL-2, or TNF was measured after stimulation with PMA/ionomycin and brefeldin A for the indicated duration. Gag^+^ cells were categorized by CD69 expression (n = 6). (Q) Mean proportion of Gag^+^ resting memory T cells expressing one, two, or three of the cytokines IFN-γ, IL-2, or TNF after 6 h of PMA/ionomycin stimulation in the presence of brefeldin A, categorized by CD69 expression (n = 8). All measurements were made after 72 h or at the indicated time post co-culture. Data are the mean ± SEM. Paired two-tailed t test or one-way ANOVA with Bonferroni or Dunnett’s post test were used. ^∗^p < 0.05; ^∗∗^p < 0.01; ^∗∗∗^p < 0.001; n.s., not significant. MFI, median fluorescence intensity.

**Figure 3:**
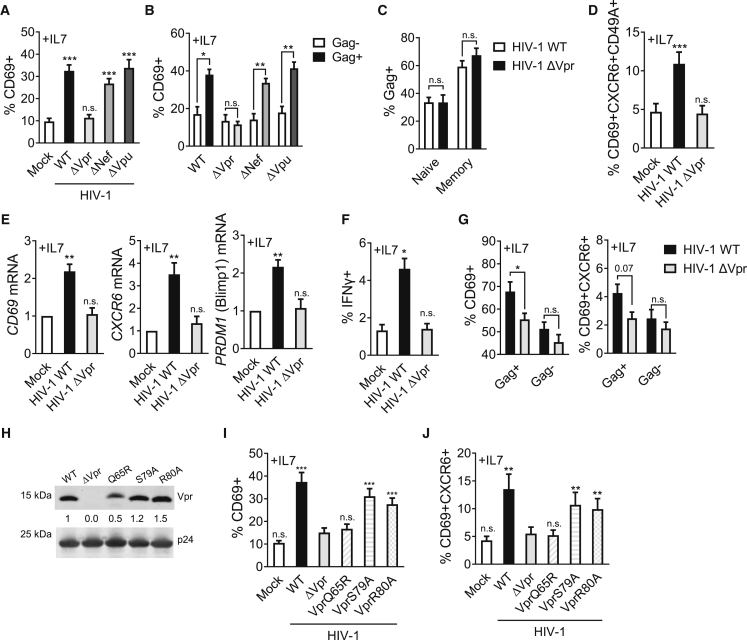
Vpr drives HIV-1-induced T_RM_ induction in resting memory CD4^+^ T cells Resting memory CD4^+^ T cells were co-cultured with primary CD4^+^ T cells infected with HIV-1 wild-type (WT) or mutant viruses or with uninfected donor cells (mock). (A) CD69 upregulation in response to IL-7 compared with mock (n = 9). (B) CD69 expression on HIV-1-infected Gag^+^ resting memory CD4^+^ T cells compared with uninfected Gag^−^ bystander cells (n = 9). (C) Quantification of cell-to-cell spread of HIV-1 WT and ΔVpr to resting naive and memory CD4^+^ T cells (n = 9). (D) CD69/CXCR6/CD49a co-expression on resting memory CD4^+^ T cells infected with HIV-1 WT or ΔVpr (n = 9). (E) *CD69*, *CXCR6*, and *PRDM1* (Blimp1) mRNA levels from FACS-sorted infected resting memory CD4^+^ T cells. Fold change relative to uninfected (mock) is shown (n = 5). (F) IFN-γ expression by HIV-1-infected resting memory CD4^+^ T cells at 72 h in response to IL-7 (n = 3). (G) Total lymphocytes from cellularized tonsils were co-cultured with HIV-1 WT- or ΔVpr-infected Jurkat T cells in the presence of IL-7. Expression of CD69 (left) or CD69/CXCR6 (right) was measured in Gag^+^ infected and Gag^−^ uninfected bystander cells (n = 4). (H) Western blot showing Vpr packaging into HIV-1 WT and Vpr-mutant virions. Values indicate Vpr levels normalized to p24, relative to WT. (I) CD69 upregulation in response to IL-7 on resting memory CD4^+^ T cells infected with HIV-1 WT, ΔVpr, or Vpr mutants (n = 9). (J) Co-expression of CD69 and CXCR6 from (I) (n = 9). All measurements were made after 72 h or at the indicated time post co-culture. Data are the mean ± SEM. Paired two-tailed t test or one-way ANOVA with Bonferroni or Dunnett’s post test was used. 2LTR circles (I) were compared by unpaired one-tailed t test. ^∗^p < 0.05; ^∗∗^p < 0.01; ^∗∗∗^p < 0.001; n.s., not significant.

Infection of resting CD4^+^ target T cells by cell-to-cell spread was preferentially detected in CD45RA^−^ resting memory T cell populations rather than CD45RA^+^ naive T cells, which are both abundant in peripheral blood (Figures 1C–1E and S2A). Co-staining for CD62L confirmed that the infected CD45RA^+^ cells were mainly naive rather than T_EMRA_ (Figures S2B and S2C) (Sallusto et al., 1999). The preferential infection of CD45RA^−^ memory T cell populations rather than the CD45RA^+^ naive population is in agreement with HIV-1 being predominantly detected in memory CD4^+^ T cells *in vivo* (Brenchley et al., 2004; Chomont et al., 2009; Shan et al., 2017). This was not due to competition between naive and memory cells, because the same effect was observed when CD45RA^+^ and CD45RA^−^ resting CD4^+^ target T cells were separated prior to cell-to-cell infection (Figures 1F and S2D). Of note, when measuring infection only in the permissive resting memory T cell population, cell-to-cell spread resulted in up to 60% infection (Figures 1D–1F). As expected this is much higher than observed in the total resting T cell population (Figure 1A), in which the analysis did not distinguish between naive (non-permissive) and memory (permissive) CD4^+^ target T cells. Cell-to-cell infection of resting memory T cells was observed with the CXCR4-tropic strain NL4.3 and CCR5-tropic viruses NL4.3 BaL and two transmitter-founder (T/F) primary isolates (CH040 and CH077) (Figures 1G–1I and S2E), demonstrating that increased permissivity was not unique to a particular virus or receptor tropism. Preventing viral entry with a fusion inhibitor (T20), or blocking reverse transcription (efavirenz), inhibited the appearance of Gag^+^ and also GFP^+^ cells (the latter using a replication-competent GFP-reporter virus) (Figures 1J, 1K, and S2F–S2I), demonstrating that this signal reflects productive infection and not simply virus capture (Hübner et al., 2009; Jolly et al., 2004; Len et al., 2017; Sourisseau et al., 2007). Consistent with productive infection, we also observed downregulation of CD4 expression on target cells that was most pronounced in the resting memory T cell population (Figures 1L, S2J, and S2K). To confirm that productively infected resting memory T cells can make new virus and propagate viral spread, we recovered HIV-1-infected resting memory target cells from co-cultures by flow sorting (Figures S1M and S2L) and returned them into culture without activation to measure infected cells and virus output over time. This confirmed that these cells supported viral replication by (1) spreading infection within the resting T cell population as evidenced by an increase in the number of Gag-positive resting T cells over time (Figure 1M), (2) producing new virus that was detected in culture supernatants (Figure 1N), and (3) transmitting infection to fresh target cells added to the culture (Figure 1O). Activating the recovered resting memory T cells by T cell receptor (TCR) cross-linking further boosted virus production, confirming that the cells remained responsive to stimuli (Figures S2M and S2N). Interestingly, resting T cells infected by cell-to-cell spread were also longer lived than their matched activated counterparts and showed improved persistence after restimulation with αCD3/αCD28 (Figures S1K and S1L), suggesting these cells may contribute to the establishment of a longer-lasting infected T cell reservoir. Collectively, these data demonstrate that cell-to-cell spread drives productive infection of resting CD4^+^ T cells that have the capacity to disseminate infection.

### HIV-1 infection induces resting memory CD4^+^ T cells to gain characteristics of tissue-resident T cells by synergizing with interleukin-7

We confirmed that HIV-1^+^ target T cells infected by cell-to-cell spread maintained their resting phenotype and did not upregulate Ki67 or MCM2, two markers of cell-cycle progression (Figures S3A and S3B). Therefore these cells are not simply being activated and driven into the cell cycle by either infection or bystander effects during co-culture. Intriguingly, expression of CD69 on HIV-1-infected resting memory target T cells was significantly increased compared with mock-treated (uninfected) target T cells (Figures 2A and 2B). Importantly, this was not due to preferential infection of a minor pre-existing population of CD69^+^ CD4^+^ T cells in blood (Figure S1C). We confirmed this by removing the small proportion of CD69^+^ blood T cells by flow cytometry sorting to recover pure CD69^−^ CD4^+^ T cells, co-culturing these cells with HIV-1-infected donor T cells, and observing *de novo* upregulation of CD69 on the newly infected resting memory target cells (Figures S3C and S3D). While CD69 is classically thought of as a marker of early T cell activation, expression can occur independent of cell-cycle progression and T cell activation (Corneau et al., 2017; Lea et al., 2003; Szabo et al., 2019b). Consistent with this, we did not detect activation concomitant with CD69 upregulation in HIV-1-infected resting CD4^+^ memory T cells, and CD69^+^ cells remained HLA-DR negative (Figure S3E).

Functionally, CD69 is crucial for T cell retention in tissues by interfering with S1P receptor-mediated egress and has been identified as a hallmark of tissue-resident memory (T_RM_) T cells (Kumar et al., 2017). Recently it has been demonstrated that although T_RM_ cells are largely absent from peripheral blood (Figure S3F), precursor cells poised to adopt a T_RM_ phenotype are present in the circulation (Almeida et al., 2022; Fonseca et al., 2020; Kok et al., 2020). Interestingly, while HIV-1 infection alone induced upregulation of CD69, additional exposure of these HIV-1-infected resting memory T cells to the homeostatic T cell cytokine interleukin-7 (IL-7) further boosted CD69 upregulation 4-fold, compared with HIV-1 or IL-7 alone (Figures 2C and S3J). IL-7 secreted by stromal cells is required for long-term maintenance of CD4^+^ T_RM_ cells (Adachi et al., 2015; Amezcua Vesely et al., 2019; Yeon et al., 2017). Although IL-7 can enhance HIV-1 infection of T cells (Coiras et al., 2016) (Figure S3G), infection of resting memory T cells mediated by cell-to-cell spread does not require IL-7 (Figure 1); furthermore, IL-7 increased CD69 expression on infected cells even when added 48 h post-infection (Figure S3J). HIV-1-induced CD69 upregulation was abrogated by suppressing infection with the fusion inhibitor T20 (Figure 2C) or by treating cells with ruxolitinib, which blocks IL-7-mediated JAK-STAT signaling (Figure 2D), demonstrating that the enhanced CD69 induction requires both infection and cytokine signaling. Similar enhancement of CD69 expression on HIV-1-infected resting cells was also observed in response to the γ_c_-chain cytokine IL-15 (Figures 2E and 2F), but not IL-12 or TGF-β (Figures S3H and S3I). Alongside CD69, resting memory T cells also increased co-expression of the T_RM_ marker CXCR6 (Kumar et al., 2017) during HIV-1 infection (Figures 2G, 2H, S3K, and S3N). Similarly, we also observed an increase in the population of CD69^+^ cells co-expressing CD49a, PD-1, and CD101, thus generating a population of resting memory T cells co-expressing multiple T_RM_ marker proteins that are associated with the defined core T_RM_ phenotypic signature (Kumar et al., 2017) (Figures 2I, 2J, and S3L). This suggests that HIV-1 infection primes CD4^+^ T cells to gain characteristics that are associated with T_RM_ cells and adopt a T_RM_-like phenotypic signature. Similar to *ex vivo* T_RM_ cells, we saw no upregulation of CX3CR1 expression (Figure S3O) and no transcriptional upregulation of *S1PR1* or *KLF2* by RT-PCR (Figure S5I); however, comprehensive RNA-sequencing (RNA-seq) analysis revealed a 2-fold downregulation of *KLF2* and *S1PR*1 in HIV-1-infected compared with uninfected (mock) cells (Data S1), consistent with their suppression under conditions of T_RM_ induction (Kumar et al., 2017). Critically, induction of the T_RM_-like phenotypic markers did not occur in uninfected Gag^−^ bystander cells (Figures 2K, S3M, and S3N). By contrast to CD8^+^ T_RM_ cells, CD103 was barely detectable and not upregulated (Figures S3P and S3Q), consistent with the observation of limited CD103 expression on CD4^+^ T cells (Kumar et al., 2017). Induction of CD69 expression was also concomitant with upregulation of the T_RM_-associated transcription factor Blimp-1 (Hombrink et al., 2016; Mackay et al., 2016; Pallett et al., 2017) (Figures 2L and 2M). Similar upregulation of T_RM_-associated markers was also observed when unstimulated CD4^+^ T cells from tonsil (Figures 2N, 2O, and S4A) or mediastinal lymph nodes (Figures S4B and S4C) were infected with HIV-1 via cell-to-cell spread and exposed to IL-7, demonstrating that induction of this T_RM_-like phenotype occurs in tissue-derived T cells following HIV-1 cell-mediated infection.

Functionally, T_RM_ cells are poised for rapid production of cytokines (IFN-γ, IL-2, and TNF) following antigenic-stimulation (Christo et al., 2021; FitzPatrick et al., 2021; Wiggins et al., 2021). Notably, we found that HIV-1-induced T_RM_-like cells produced these cytokines faster and to higher levels following recall stimulation compared with HIV-1-infected CD69^−^ non-T_RM_ cells (Figure 2P) and also showed a greater propensity to produce multiple cytokines per cell (Figure 2Q). Taken together, these data suggest that HIV-1 infection of resting memory CD4^+^ T cells reprograms cells by upregulating expression of a combination of cell-surface proteins and inducing T cells to gain phenotypic and functional characteristics that are associated with tissue residency, which we term “T_RM_-like” cells.

### Vpr is required for induction of the T_RM_-like phenotype in HIV-1-infected T cells

HIV-1 expresses four accessory proteins, Vif, Vpu, Vpr, and Nef, which directly and indirectly manipulate host cell factors to facilitate efficient viral replication *in vivo* and drive pathogenesis (Malim and Emerman, 2008). Co-culture of resting target T cells with donor T cells infected with HIV-1 accessory protein mutants showed that deletion of Vpr (HIV-1 ΔVpr) prevented induction of the T_RM_-like phenotype following HIV-1 infection, evidenced by no CD69 upregulation and no increase in the CD69^+^/CXCR6^+^/CD49a^+^ triple-positive T_RM_-like memory population (Figures 3A, 3B, 3D, S5A–S5E, and S6). By contrast, deletion of Vpu or Nef did not affect HIV-1 induction of these marker proteins on infected resting T cells (Figures 3A, 3B, S5A–S5E, and S6). HIV-1 ΔVif could not be tested because Vif is required to antagonize APOBEC3-mediated viral restriction and allow infection (Sheehy et al., 2003). HIV-1 Vpr is not required for infection of T cells *in vitro* (Balliet et al., 1994; Rogel et al., 1995), and concordantly, lack of changes to T_RM_-marker protein expression was not due to lack of infection of resting target cells by HIV-1 ΔVpr virus nor reduced Gag expression (Figures 3C, S5F, S5G, and S5H). Like WT virus, ΔVpr virus also maintained a preferential tropism for resting memory T cells over naive T cells (Figure 3C). Critically, Vpr was required for induction of CD69 expression observed at the mRNA level, as well as induction of *CXCR6* and Blimp1 (*PRDM1*) mRNA (Figure 3E). As expected, there was no upregulation of *S1PR1* or *KLF2* mRNA by either HIV-1 WT or ΔVpr virus (Figure S5I). Vpr also mediated spontaneous production of IFN-γ by infected CD4^+^ memory T cells (Figure 3F), characteristic of T_RM_ cells (FitzPatrick et al., 2021; Wiggins et al., 2021). Importantly, Vpr-dependent changes were also observed using tonsil-derived tissue memory T cells as targets for cell-to-cell spread (Figures 3G, S5K, and S5L). While a proportion of tonsil-derived target cells already express residency markers at baseline as expected, expression was further increased on WT HIV-1-infected cells but not ΔVpr-infected cells (Figures 3G, S5K, and S5L), confirming the requirement for Vpr in driving T_RM_-like phenotypic changes in tissue cells *ex vivo*.

Vpr is a multifunctional protein that is packaged into viral particles and is present during the early stages of infection, in which it plays an important, but as yet poorly defined, role in HIV-1 pathogenesis. Among the best-defined functions of Vpr are its ability to (1) bind the Cul4A-DDB1 (DCAF1) complex, leading to an interaction with the ubiquitinylation and proteasomal machinery; (2) induce G2/M cell-cycle arrest; and (3) drive apoptosis in infected cells (Jowett et al., 1995; Laguette et al., 2014; Schröfelbauer et al., 2007; Wen et al., 2007; Wu et al., 2016). We abrogated these functions individually by introducing the Vpr mutations Q65R, S79A, or R80A, respectively, into HIV-1 and confirmed that each Vpr mutant is packaged into virions (Figure 3H). Co-culture of resting memory target T cells with HIV-1^+^ T cells infected with different Vpr mutants revealed that the cell-cycle arrest mutants S79A and R80A behaved similar to WT virus and induced CD69 and CXCR6 upregulation (Figures 3I, 3J, and S5J). By contrast, Q65R, which is most closely associated with loss of DCAF1 binding, was unable to induce a T_RM_-like phenotype following infection of target cells, behaving like ΔVpr virus in these experiments (Figures 3I, 3J, and S5J).

### Vpr packaged into incoming virions mediates the T_RM_-like phenotype

**Figure 4:**
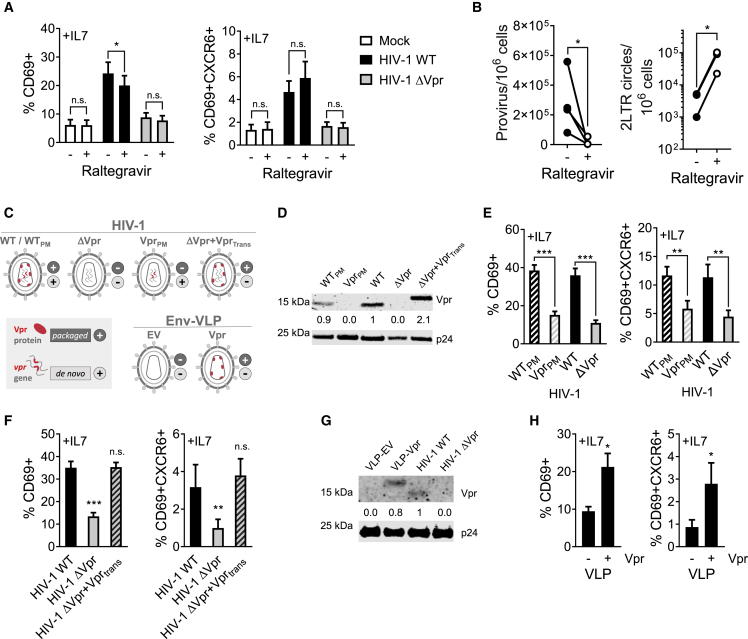
Incoming Vpr is sufficient to drive T_RM_ induction in resting memory CD4^+^ T cells (A) CD69 (left) and CD69/CXCR6 (right) co-expression in response to IL-7 in the presence of integrase inhibitor raltegravir (n = 6). (B) Quantification of integrated provirus and 2LTR circles in FACS-sorted target CD4^+^ memory T cells after 72 h of cell-to-cell spread in the presence or absence of raltegravir. (C) Schematic depicting the viruses and VLPs used in (D–H). (D) Western blot showing Vpr packaging into virions of HIV-1 WT_PM_, Vpr_PM_, WT, ΔVpr, and ΔVpr complemented with FLAG-tagged Vpr *in trans* (ΔVpr^+^Vpr_t__rans_). (E) CD69 (left) and CD69/CXCR6 (right) upregulation in response to IL-7 on Gag^+^ resting memory CD4^+^ T cells at 72 h infected with the indicated HIV-1 viruses (n = 8). (F) Expression of CD69 (left) and CD69/CXCR6 (right) in response to IL-7 on Gag^+^ resting memory CD4^+^ T cells at 72 h post spinoculation of HIV-1 WT, Vpr, and ΔVpr^+^Vpr_trans_ (n = 10). (G) Western blot showing packaging of FLAG-tagged Vpr into Env-VLPs or full-length HIV-1 WT or ΔVpr. (H) Expression of CD69 (left) and CD69/CXCR6 (right) in response to IL-7 on Gag^+^ resting memory CD4^+^ T cells at 72 h post spinoculation of Env-VLPs with or without Vpr (n = 5). All measurements were made after 72 h or at the indicated time post co-culture or spinoculation. Data are the mean ± SEM. Paired two-tailed t test or one-way ANOVA with Bonferroni or Dunnett’s post test was used. ^∗^p < 0.05; ^∗∗^p < 0.01; ^∗∗∗^p < 0.001; n.s., not significant. EV, empty vector.

Vpr-mediated induction of a T_RM_-like phenotype was not inhibited by potently suppressing HIV-1 integration into resting memory CD4^+^ T cells using the integrase inhibitor raltegravir (Figures 4A and 4B). The presence of integrated provirus in resting CD4^+^ T cells at approximately one provirus per infected cell (Figure 4B) supports the observation that highly efficient cell-to-cell spread results in resting CD4^+^ T cell infection (Figure 1), but notably without leading to multiple proviral integrations. Vpr is packaged into HIV-1 virions and as such is delivered into the target cell during the earliest steps of infection. To determine whether incoming virion-associated Vpr was sufficient to drive the phenotypic changes in the presence of IL-7, we took three approaches (Figure 4C). First, we used an HIV-1 mutant in which the Gag-p6 Vpr packaging sequence was mutated to prevent Vpr incorporation into virions (Radestock et al., 2013; Wanaguru and Bishop, 2021) (Figures 4D and 4E). This mutant (which we termed Vpr_PM_) cannot package Vpr into virions, but retains an intact *vpr* gene, allowing for *de novo* Vpr synthesis from the viral genome following infection, and maintains the capacity for cell-to-cell spread (Figure S7A). Preventing Vpr packaging into virions abrogated upregulation of CD69 and CXCR6 on resting memory T cells compared with the parental WT control (termed WT_PM_) (Figure 4E). Second, complementing the ΔVpr virus with Vpr *in trans* allowed for Vpr packaging into virions and delivery into cells without *de novo* Vpr synthesis during infection, and fully rescued upregulation of CD69 and CXCR6 spinoculation of resting CD4^+^ T cells (Figures 4D, 4F, S7B, and S7C). Finally, we made infectious but non-replicative HIV-1 Env pseudotyped virus-like particles (Env-VLPs) that do not contain an HIV-1 genome but can package Vpr expressed *in trans* during VLP production (Env-VLP-Vpr), and confirmed sufficient Vpr delivery for degradation of a known Vpr target, UNG2, upon spinoculation (Figures S7D, S7E, and S7F). Env-VLPs allow efficient particle entry into resting CD4^+^ T cells, which are not permissive to VSVg-mediated transduction. Using Env-VLP-Vpr particles to deliver Vpr in the absence of viral replication was sufficient to induce co-expression of CD69 and CXCR6 in the presence (Figures 4G and 4H), but not in the absence (Figure S7G), of IL-7. Taken together, we conclude that incoming Vpr in virions is sufficient to drive T cells to upregulate T_RM_ markers in synergy with IL-7.

### Vpr induces widespread transcriptional reprogramming in HIV-1-infected resting T cells and a core T_RM_ transcriptional signature

**Figure 5:**
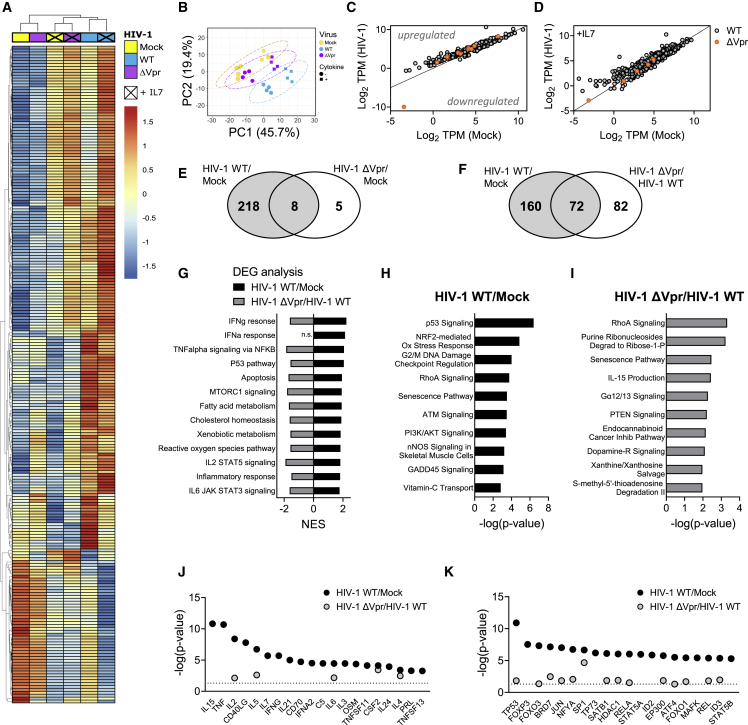
Transcriptional profiling of HIV-1-infected resting memory CD4^+^ T cells (A) Heatmap showing hierarchical clustering of 226 differentially expressed genes (DEGs) of infected (HIV-1 WT) over uninfected (mock) resting memory CD4^+^ T cells at 72 h post co-culture (adjusted p < 0.01, fold change ±1.2). Mean log2 TPM of four biological repeats are shown. Cytokine indicates presence or absence of IL-7. Virus indicates infection with HIV-1 WT, HIV-1 ΔVpr, or uninfected (mock) condition. (B) Principal-component analysis (PCA) of (A), with ellipses indicating 95% CI. (C and D) Scatterplots of mean log2 TPMs of DEGs from HIV-1 WT/mock (gray circles) or HIV-1 ΔVpr/mock (orange circles) in the absence (C) or presence (D) of IL-7 (adjusted p < 0.01, fold change ±1.2). Lines indicate line of identity (LOD). Genes above or below the LOD are up- or downregulated, respectively. (E and F) Venn diagrams showing overlap of DEGs comparing expression profiles of HIV-1 WT/mock with HIV-1 ΔVpr/mock (E) or HIV-1 ΔVpr/HIV-1 WT (F). (G) GSEA was performed on expression profiles comparing HIV-1 WT/mock (black) or HIV-1 ΔVpr/HIV-1 WT (gray). Normalized enrichment scores (NES) are shown for significantly enriched hallmark gene sets (false discovery rate [FDR] q < 0.05 and NES > 1.75). (H and I) Top 10 significantly enriched canonical pathways predicted by ingenuity pathway analysis (IPA) of DEGs in HIV-1 WT/mock (H) or HIV-1 ΔVpr/HIV-1 WT (I) (adjusted p < 0.05). (J and K) Cytokines (J) and transcription regulators (K) predicted to be upstream regulators by IPA of gene expression signatures for HIV-1 WT/mock (black) or HIV-1 ΔVpr/mock (gray); line indicates p = 0.05. TPM, transcripts per million.

Next we performed transcriptional profiling by RNA-seq analysis of flow-cytometry-sorted resting memory CD4^+^ T cells infected with HIV-1 WT or ΔVpr virus by cell-to-cell spread in the presence and absence of IL-7. HIV-1 infection alone induced widespread changes in gene expression in resting memory T cells compared with uninfected cells (226 differentially expressed genes [DEGs], fold change >1.2, adjusted p < 0.01) (Figure 5). Hierarchical clustering and principal-component analysis revealed that the gene expression patterns in response to HIV-1 WT infection were clearly distinct from those induced following infection with ΔVpr virus (Figures 5A–5C, 5E, and 5F) demonstrating that *Vpr* deletion suppresses the global transcriptional response to HIV-1 infection. Specifically, infection with ΔVpr virus resulted in only 13 genes showing statistically significant changes compared with uninfected cells, in contrast to 226 genes for HIV-1 WT virus (Data S1; Figure 5E). In fact, much of the transcriptional response to HIV-1 was regulated by Vpr, as evidenced by striking differences in the number of DEGs in the presence and absence of Vpr (Figures 5E and 5F). The requirement for Vpr in driving many of the changes in DEGs was also observed when infected cells were exposed to IL-7, implicating the virus as the dominant driver of T cell reprogramming in our experiments (Figures 5A, 5B, 5D, and S8; Data S1).

To gain greater insight into the native effects of HIV-1 and Vpr on T cells, gene set enrichment analysis (GSEA) (Figure 5G) and ingenuity pathway analysis (IPA) (Figures 5H and 5I) were performed on data from infected cells in the absence of IL-7 treatment, since IL-7 was not required for productive infection of resting memory T cells (Figure 1). Consistent with Vpr manipulating the T cell response to HIV-1 infection (Data S1), these data revealed enrichment of numerous cellular signaling pathways following HIV-1 infection that appeared Vpr dependent, most notably, pathways associated with cytokine and inflammatory responses as well as immune signaling (Figures 5G and S8; Data S1). This was further evidenced by upstream regulator analysis that showed significant enrichment for genes associated with cytokine signaling and transcriptional regulators that were again largely Vpr dependent (Figures 5J, 5K, and S8). For comparison the same analysis of HIV-1 WT and ΔVpr virus-infected cells in the presence of IL-7 is shown (Figure S8). Taken together, these data reveal that HIV-1 induces dramatic reprogramming during infection of resting memory CD4^+^ T cells driven largely by Vpr.

**Figure 6:**
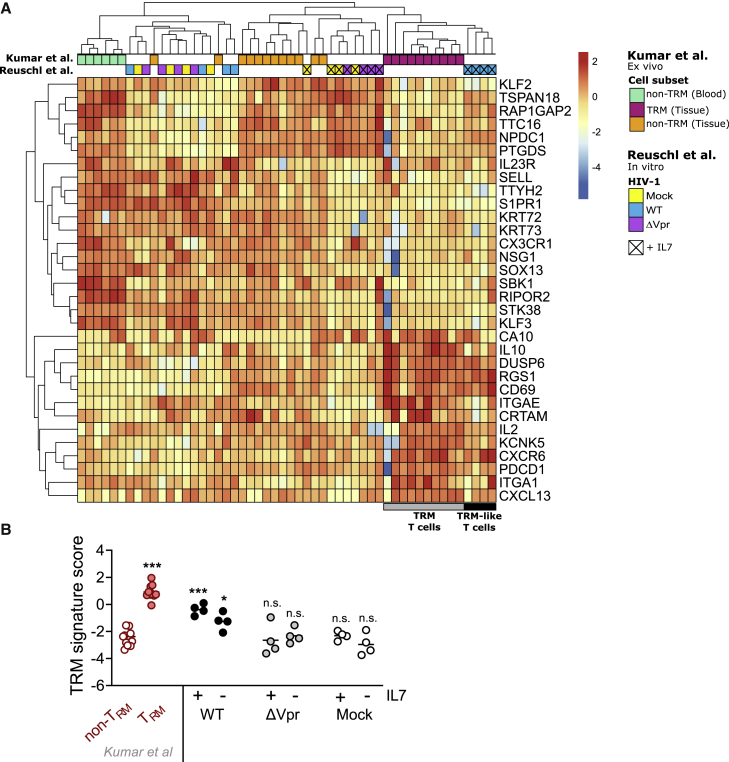
Vpr drives a T_RM_-like transcriptomic program in HIV-1-infected resting memory CD4^+^ T cells (A) Heatmap showing hierarchical clustering based on a T_RM_ core gene expression signature (Kumar et al., 2017) that was performed to compare transcriptional profiles of *in vitro* HIV-1-infected resting memory CD4^+^ T cells (mock, HIV-1 WT, HIV-1 ΔVpr) with previously described *ex vivo* gene expression profiles (Kumar et al., 2017). Kumar et al. “Cell subset” indicates *ex vivo* CD69^+^ T_RM_ (TRM [tissue]), CD69^−^ non-T_RM_ (non-TRM [tissue]), tissue-derived T cells (from lung or spleen), and blood-derived CD69^−^ T cells (non-TRM [blood]). Reuschl et al. “HIV-1” indicates infection with HIV-1 WT, HIV-1 ΔVpr, or uninfected (mock) conditions. Presence of IL-7 is indicated by X. (B) The T_RM_ signature score for the indicated conditions calculated based on (A). Subsets from Kumar et al. (2017) are indicated in red; shown are CD4^+^ or CD8^+^ T cells from lungs or spleens. T_RM_^+^, CD69^+^ T cells; T_RM_^−^, CD69^−^ T cells. T_RM_ signature scores for resting CD4^+^ memory T cells infected or uninfected are shown in the presence or absence of IL-7. Means are shown. One-way ANOVA with Dunnett’s post test was used to compare groups in (B).

Tissue residency of T cells has been associated with a 31-gene core transcriptional signature (Kumar et al., 2017). We took advantage of this dataset and our RNA-seq analysis to determine whether this core T_RM_ gene signature was enriched in our transcriptome of HIV-1-infected resting memory T cells. Hierarchical clustering of our data comparing with the core gene signature of T_RM_ cells (CD69^+^ T cells isolated from human lung and spleen) (Kumar et al., 2017) showed that HIV-1-infected memory T cells exposed to IL-7 were grouped distinctly and clustered with *bona fide* CD69^+^ T_RM_ cells (Figure 6A; Data S1) and were distinct from non-T_RM_ T cells (CD69^−^ T cells isolated from tissue and blood from Kumar et al., 2017). We further corroborated this finding by calculating a T_RM_ gene enrichment score, using the gene expression data in the published core T_RM_ transcriptional signature. Extracting this 31-gene set from our RNA-seq data and performing a statistical comparison of gene expression between our data and that of Kumar et al. (2017) showed that HIV-1-infected resting memory T cells (±IL-7) harbor a T_RM_ signature score (and thus gene expression profile) that approximated closely with that of *bona fide* T_RM_ cells (Figure 6B) and was statistically significantly different from non-T_RM_ T cells. Critically, this was Vpr dependent, with mock- and ΔVpr-infected cells showing an enrichment score that was more closely aligned with and not statistically different from non-T_RM_. Thus, we conclude that HIV-1 Vpr induces resting T cells to gain a constellation of features that define T_RM_ cells, including co-expression of key phenotypic T_RM_ surface markers, functional characteristics associated with T_RM_ T cell recall responses, and, as our comprehensive RNA-seq reveals, a transcriptional T_RM_-like signature via the accessory protein Vpr.

### Vpr activates STAT5 to synergize with IL-7 signaling for T cell reprogramming

**Figure 7:**
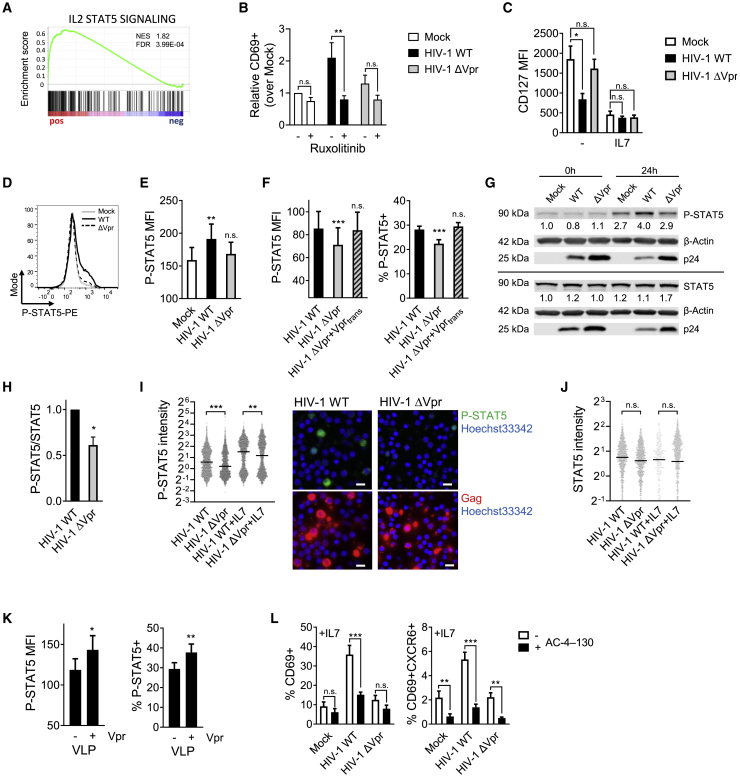
Vpr enhances STAT5 activation to drive a T_RM_-like resting memory CD4^+^ T cell phenotype (A) GSEA enrichment plot of the hallmark IL-2 STAT5 signaling pathway for HIV-1 WT-infected resting memory T cells versus mock. (B) CD69 expression on infected resting memory CD4^+^ T cells ± ruxolitinib at 72 h (n = 4). (C) CD127 MFI on infected resting memory CD4^+^ T cells ± IL-7 (n = 7) at 72 h. (D) Representative histogram of intracellular STAT5 phosphorylation in resting memory T cells infected by cell-to-cell spread at 72 h. (E) Quantification of (D) shown as P-STAT5 MFI (n = 10). (F) P-STAT5 MFI (left) and %P-STAT5^+^ (right) in Gag^+^ resting memory T cells 24 h post spinoculation with the indicated viruses (n = 12). (G) Representative western blot analysis of P-STAT5 and total STAT5 levels in resting T cells at 0 and 24 h post spinoculation with HIV-1 WT and ΔVpr virus (n = 2). Values indicate P-STAT5 or total STAT5 levels normalized to β-actin and mock at 0 h. (H) Quantification of P-STAT5/STAT5 levels normalized to β-actin from western blots of total CD4^+^ T cells at 24 h post spinoculation with HIV-1 WT and ΔVpr virus (n = 4). (I and J) Quantification of (I) P-STAT5 and (J) total STAT5 levels by single-cell immunofluorescence analysis of Gag^+^ resting T cells 24 h post spinoculation with HIV-1 WT and ΔVpr virus ± IL-7 (P-STAT5 n = 1,829–2,000 cells/condition; STAT5 n = 223–900 cells/condition). Normalized mean intensities (quantifications) and representative images of P-STAT5 in HIV-1 WT- and ΔVpr-infected cells without IL-7 (I, right) are shown. P-STAT5, green; Gag, red; Hoechst 33342, blue. Scale bars, 10 μm. (K) P-STAT5 MFI and %P-STAT5^+^ (right) in Gag^+^ resting memory T cells 24 h post spinoculation with VLPs with or without Vpr (n = 7). (L) CD69 (left) and CD69/CXCR6 (right) expression on infected resting memory CD4^+^ T cells in the presence of IL-7 ± STAT5-inhibitor AC-4-130 at 72 h (n = 6). All measurements were made after 72 h or at the indicated time post co-culture or spinoculation. Data are the mean ± SEM. Paired two-tailed t test or one-way ANOVA with Bonferroni or Dunnett’s post test was used. For (I) and (J), median is indicated and groups were compared using Kruskal-Wallis test with Dunn’s post test. ^∗^p < 0.05; ^∗∗^p < 0.01; ^∗∗∗^p < 0.001; n.s., not significant. MFI, median fluorescence intensity.

Having shown that HIV-1 Vpr drives a T_RM_-like phenotype in resting memory T cells and primes cells to become hyperresponsive to IL-7, we hypothesized that Vpr may do so by manipulating JAK-STAT signaling, the pathway downstream of IL-7 signals. In support of this, GSEA of our RNA-seq data revealed increased expression of genes regulated by the transcription factor STAT5 (Figures 5G, 7A, and S8C), including *CD69* (Kanai et al., 2014), as well as enrichment of common-gamma-chain cytokine signaling (Figures 5 and S8), consistent with the hypothesis that HIV-1 manipulates STAT5 in resting memory T cells. Furthermore, HIV-1-dependent upregulation of the T_RM_ marker CD69 was inhibited by ruxolitinib treatment, which blocks JAK-STAT signaling, in a Vpr-dependent manner (Figure 7B). Testing this further, we found that infection also downregulated the IL-7 receptor α subunit (CD127) from the cell surface (Figure 7C) and transcriptionally (fold change = 0.693, adjusted p = 5.89 × 10^−8^; Data S1) (Park et al., 2004), collectively indicative of HIV-1 inducing enhanced JAK-STAT signaling even in the absence of IL-7. Importantly, Vpr also mediated STAT5-activation during HIV-1 cell-to-cell infection of resting memory CD4^+^ T cells as evidenced by an increase in the intracellular levels of phosphorylated STAT5 (P-STAT5) (Figures 7D and 7E) under conditions where cells were not exposed to IL-7. A similar, Vpr-dependent increase in P-STAT5 was also seen when Vpr was delivered into resting T cells by spinoculation of infectious HIV-1 (Figures 7F–7J) or using Env-Vpr-VLPs (Figure 7K), confirming again that incoming Vpr is sufficient to drive reprogramming of resting memory T cells. Given that STAT5 drives *CD69* gene expression, we next treated resting memory T cells with the selective STAT5 inhibitor AC-4-130 (Wingelhofer et al., 2018). Strikingly, blocking STAT5 activity in this way completely inhibited induction of the T_RM_ phenotype by HIV-1 infection, preventing upregulation of CD69 and CXCR6 (Figures 7L and S5O). AC-4-130 inhibited CD69 upregulation by HIV-1 Vpr in both the absence and the presence of IL-7, consistent with HIV-1 alone activating STAT5 via Vpr, resulting in increased CD69 expression. Taken together, these data suggest a mechanism by which Vpr manipulates cellular signaling pathways and STAT5 phosphorylation, making T cells hyperresponsive to IL-7, which works in synergy with HIV-1 to drive induction of a T_RM_-like phenotype (Figure 7I).

## Discussion

Our discovery that resting CD4^+^ T cells can be productively infected by cell-to-cell spread, allowing for viral integration, replication, and dissemination, transforms our ability to determine how T cells respond to and support HIV-1 replication without confounding activation-induced changes. Here, employing our co-culture model, we have revealed that HIV-1 infection of resting memory T cells induces T cell reprogramming, resulting in these cells gaining characteristics that are associated with T_RM_ T cells, a phenotype that we have termed T_RM_-like. This is evidenced by the upregulation and co-expression of T_RM_-associated marker proteins on infected cells (e.g., CD69/CXCR6/CD49a triple-positive cells), induction of a core T_RM_ transcriptional signature, and the gain of functional characteristics associated with T_RM_ cells. HIV-1 establishes cellular and tissue reservoirs, both active and latent, that ultimately prevent cure with anti-retroviral therapy. Importantly, T_RM_ cells are long-lived and are thought to be largely confined to tissue (Kumar et al., 2018), providing an alternate model for a tissue-associated reservoir driven by the virus itself. Our results suggest that HIV-1 persistence and the establishment of tissue reservoirs may be driven, in part, through direct viral induction of a T_RM_-like phenotype via transcriptional reprogramming. Recently, T_RM_ cells in cervical tissue were found to be preferentially infected by HIV-1 and can harbor an HIV-1 reservoir *in vivo* (Cantero-Pérez et al., 2019). The relative contribution of pre-existing versus HIV-1-induced T_RM_ cells to viral reservoirs and their relative abundance in different anatomical compartments *in vivo* remain to be quantified, but we expect T_RM_ cells harboring virus to be important contributors to viral persistence. In light of these findings it is possible that HIV-1-infected cells circulating in peripheral blood may in fact represent cells that have failed to become part of the tissue reservoir, leading to an underestimation of the true viral burden. Having shown that HIV-1 infection of resting T cells by cell-to-cell spread results in productive infection, while being less sensitive to HIV-1-mediated cytotoxicity, we hypothesize that induction of a T_RM_-like phenotype in infected cells may also play additional roles in establishing and maintaining viral reservoirs by sequestering infected cells in tissue sites where susceptible target T cells are in abundance, thus supporting localized viral replication and spread. Indeed we have shown that infected resting memory T cells support spreading infection to disseminate virus. Given the importance of T_RM_ cells as a population that is increasingly recognized to be critical in providing localized immunity and immunosurveillance (Pallett et al., 2017; Szabo et al., 2019b), future work should focus on understanding the contribution of HIV-1-induced T_RM_-like cells in pathogenesis and persistence. While HIV-1 infection directly reprograms T cells to gain core features shared across human T_RM_ populations *in vivo*, these virus-induced cells might still reveal functional differences from the host’s various subsets of “native” CD4^+^ T_RM_ cells. Dissecting and comparing these further to better understand the causes and consequences for T cell fate and function will be important. More broadly, it is now emerging that committed T_RM_ precursors, imprinted with the capacity to become mature T_RM_, pre-exist in blood and that, when exposed to the appropriate cues in tissues or *ex vivo*, can become tissue-resident (Fonseca et al., 2020; Kok et al., 2020). In fact, studies of long-term chimerism after hematopoietic transplantation have recently further confirmed this in humans (Almeida et al., 2022). Thus the ontogeny, derivation, and maintenance of T_RM_ cells, and their heterogeneity *in vivo*, appear more complex that initially appreciated. Our discovery that HIV-1 induces a T_RM_-like phenotype in CD4^+^ T cells provides an opportunity to gain new understanding of mechanisms behind CD4^+^ T_RM_ induction and maintenance.

Recently, it has been reported that cell-to-cell spread can also facilitate latent infection of resting T cells without productive infection (Agosto et al., 2018). Together, our work and that of Agosto et al. highlight the distinct advantages of co-culture models that do not require mitogenic, experimental stimulation of T cells to drive infection to study native HIV-1-host cell interactions, permissivity, and the cellular response to infection. Having shown that resting memory T cells are preferentially infected by cell-to-cell spread compared with naive cells, future studies should address how cell-to-cell spread drives permissivity and what regulates the selective permissivity of resting memory cells (for example, whether this is influenced by the expression of surface receptors involved in cell-cell spread and/or other factors downstream of viral entry) to shed new light on the interaction between HIV and host T cells.

We found that HIV-1 infection of resting memory T cells was associated with striking transcriptional reprogramming that was driven by Vpr, thus identifying a novel function for this enigmatic HIV accessory protein. Crucially, Vpr is packaged into HIV-1 virions and is thus present upon virus entry into the target cell and during the earliest events of infection. Using a series of complementary but distinct approaches, we showed that incoming Vpr is in fact sufficient to drive T_RM_ remodeling of T cells, independent of viral integration, and that Vpr induction of this phenotype does not require *de novo* Vpr transcription and protein synthesis, allowing HIV-1 to directly and immediately reshape the niche in which it resides. Vpr-mediated induction of the T_RM_-like phenotype was dependent on residue Q65, and Vpr is reported to drive widespread remodeling of the cellular proteome via its recruitment of DCAF1 through Q65 (Greenwood et al., 2019). Whether this requirement for Q65 in induction of a T_RM_-like phenotype is DCAF1 dependent remains unclear, because DCAF1 knockdown in primary T cells made cells hyperresponsive to HIV-1-induced cell death (Figures S5M and S5N). Moreover, given that Q65R did show a partial reduction in Vpr packaging, it is possible that the reduced Vpr effect is due to reduced Vpr delivery. We also cannot discount the effects of Q65R on mislocalizing Vpr (Khan et al., 2020), resulting in loss of function and thus T cell reprogramming. Thus we cannot at present formally exclude other functions of Vpr Q65 in the process of T_RM_ induction. However, our data showing that Vpr manipulates the JAK-STAT pathway through which IL-7 signals and activates STAT5 suggests a mechanism by which Vpr can work in synergy with IL-7, driving T cells to gain characteristics of T_RM_ cells.

Notably, HIV-1 induction of a T_RM_-like phenotype via Vpr was accompanied by induction of a T_RM_ transcriptional signature that aligned closely with a published core T_RM_ signature (Kumar et al., 2017). Vpr deletion abolished not only the induction of this T_RM_ signature, but also many HIV-1-induced changes to gene expression following infection of resting T cells. This is in keeping with widespread proteome remodeling by Vpr in activated T cells (Greenwood et al., 2019), but suggests that these changes may be driven in part by a hitherto unappreciated role for Vpr in modulating the host cell gene expression profile. Whether the reprogramming of resting memory T cells into T_RM_-like cells reflects widespread epigenetic changes mediated by Vpr or manipulation of key upstream regulators remains to be determined. In this regard, a recent elegant study has revealed that Vpr function is associated with epigenetic remodeling of the host cell (Dupont et al., 2021). Moreover, having found that Vpr manipulates STAT5, a crucial transcription factor for T cell function and survival, to mediate T_RM_-like phenotypic changes in resting memory T cells, it is intriguing to note that persistent STAT5 activity has been implicated in altering the epigenetic landscape of CD4^+^ T cells (Ding et al., 2020). While to date a formal role for STAT5 has not been described in T_RM_ formation, we propose that a contribution of this transcription factor to this process should be investigated further. Our data show that HIV-1 Vpr poises T cells for increased responsiveness to external stimuli by manipulation of immune signaling pathways, including innate and inflammatory responses. This is particularly intriguing and suggests that HIV-1 manipulates crucial immune signaling pathways to benefit the virus, in this case by priming resting memory T cells for T_RM_-like induction.

Notably, a rare case of laboratory-derived infection with Vpr-defective HIV-1 was characterized by markedly delayed seroconversion, suppressed viremia, and normal CD4^+^ T cell counts (Ali et al., 2018), consistent with reduced pathogenesis and failure to establish and maintain a significantly large tissue reservoir. We envisage therapeutic targeting of Vpr to manipulate persistence and pathogenesis. To achieve an HIV-1 cure, it is essential to understand the nature and establishment of HIV-1 reservoirs and how to manipulate them. By demonstrating that HIV-1 infection drives a T_RM_-like phenotype during infection of resting memory T cells, we have taken a significant step to help accelerate the quest for an HIV-1 cure.

### Limitations of the study

Here we have shown that HIV-1 infection of resting memory CD4^+^ T cells *in vitro* induces T cells to differentiate and gain characteristics that align closely with a phenotypic and transcriptional program of T_RM_ T cells. We are mindful to term these cells “T_RM_-like” T cells since no single phenotypic marker might faithfully identify a cell as tissue-resident. While differentiation of T_RM_ cells *in vitro* has been reported previously (Pallett et al., 2017; Wiggins et al., 2021), *bona fide* T_RM_ cells *in vivo* are defined by their anatomical location in tissues. Future work will be required to determine to what extent HIV-1 infection and/or expression of Vpr alone is able to induce this T_RM_-like state *in vivo* and whether HIV-1 infection reprograms resting T cells to adopt a T_RM_ profile and localization *in situ*. Currently, no appropriate *in vivo* models are available to mimic human T_RM_ biology. Thus, while these experiments will be challenging, and will necessitate the development of new humanized mouse models that allow us to capture human T_RM_ cell generation and localization while also supporting HIV-1 infection, they will be important to define to what extent this HIV-1-induced T cell reprogramming overlaps T_RM_ cells *in vivo* or reflects other T cell differentiation states.

## STAR★Methods

### Key resources table

**Table:** 

REAGENT or RESOURCE	SOURCE	IDENTIFIER
**Antibodies**		

Ultra-LEAF™ Purified anti-human CD3 Antibody (clone: OKT3)	Biolegend	Cat# 317326;RRID: AB_11150592
Ultra-LEAF™ Purified anti-human CD28 Antibody (cone: CD28.2)	Biolegend	Cat# 302934;RRID: AB_11148949
Brilliant Violet 510™ anti-human CD3 Antibody (clone: UCHT1)	Biolegend	Cat# 300448; RRID: AB_2563468
Brilliant Violet 711™ anti-human CD3 Antibody (clone: UCHT1)	Biolegend	Cat# 300464; RRID: AB_2566036
FITC anti-human CD3 Antibody (clone: UCHT1)	Biolegend	Cat# 300406; RRID: AB_314060
PE anti-human CD8 Antibody (clone: SK1)	Biolegend	Cat# 344706; RRID: AB_1953244
Brilliant Violet 605™ anti-human CD8 Antibody (clone: SK1)	Biolegend	Cat# 344742; RRID: AB_2566513
APC/Fire™ 750 anti-human CD4 Antibody (clone: SK3)	Biolegend	Cat# 344638; RRID: AB_2572097
PE/Dazzle™ 594 anti-human CD45RA Antibody (clone: HI100)	Biolegend	Cat# 304146; RRID: AB_2564079
Brilliant Violet 421™ anti-human CD45RA Antibody (clone: HI100)	Biolegend	Cat# 304130; RRID: AB_10965547
PerCP/Cyanine5.5 anti-human CD45RO Antibody (clone: UCHL1)	Biolegend	Cat# 304222; RRID: AB_2174124
Brilliant Violet 785™ anti-human CD62L Antibody (clone: DREG-56)	Biolegend	Cat# 304830; RRID: AB_2629555
APC/Fire™ 750 anti-human CD69 Antibody (clone: FN50)	Biolegend	Cat# 310946; RRID: AB_2616709
PE/Dazzle™ 594 anti-human CD69 Antibody (clone: FN50)	Biolegend	Cat# 310942; RRID: AB_2564277
PE/Dazzle™ 594 anti-human CD186 (CXCR6) Antibody (clone: K041E5)	Biolegend	Cat# 356016; RRID: AB_2563974
Anti-MCM2 antibody	Abcam	Cat# ab4461; AB_304470
PerCP/Cyanine5.5 anti-human HLA-DR Antibody (clone: L243)	Biolegend	Cat# 307630; RRID: AB_893567
PE/Dazzle™ 594 anti-human CD25 Antibody (clone: M-A251)	Biolegend	Cat# 356126; RRID: AB_2563562
PE/Cyanine7 anti-human CD38 Antibody (clone: HIT2)	Biolegend	Cat# 303516; RRID: AB_2072782
PE/Cyanine7 anti-human CD49a Antibody (clone: TS2/7)	Biolegend	Cat# 328312; RRID: AB_2566272
PE/Cyanine7 anti-human CD279 (PD-1) Antibody (clone: EH12.2H7)	Biolegend	Cat# 329918; RRID: AB_2159324
Brilliant Violet 711™ anti-human Ki-67 Antibody (clone: Ki-67)	Biolegend	Cat# 350516; RRID: AB_2563861
PE anti-human Ki-67 Antibody (clone: Ki-67)	Biolegend	Cat# 350504; RRID: AB_10660752
PE Rat Anti-Blimp-1 (clone: 6D3)	BD Biosciences	Cat# 564702; RRID: AB_2738901
PE/Cyanine7 anti-human CD101 (BB27) Antibody (clone: BB27)	Biolegend	Cat# 331013; RRID: AB_2716108
PE/Dazzle™ 594 anti-human CX3CR1 Antibody (clone: 2A9-1)	Biolegend	Cat# 341623; RRID: AB_2687151
Brilliant Violet 711™ anti-human CD103 (Integrin αE) Antibody (clone: Ber-ACT8)	Biolegend	Cat# 350221; RRID: AB_2629650
PE/Cyanine7 anti-human CD127 (IL-7Rα) Antibody (clone: A019D5)	Biolegend	Cat# 351320; RRID: AB_10897098
PE anti-human IFN-γ Antibody (clone: B27)	Biolegend	Cat# 506507; RRID: AB_315440
PE Mouse Anti-Stat5 (pY694) (clone: 47)	BD Biosciences	Cat# 612567; RRID: AB_399858
PE anti-DYKDDDDK Tag Antibody (clone: L5)	Biolegend	Cat# 637310; RRID: AB_2563148
HIV-1 core antigen-FITC (clone: KC57)	Beckman Coulter	Cat# 6604665; RRID: AB_1575987
HIV-1 core antigen-RD1 (clone: KC57)	Beckman Coulter	Cat# 6604667; RRID: AB_1575989
Antiserum to HIV-1 p24 (ARP432)	donated by Dr G. Reid and obtained from the CFAR	Cat# 0432
HIV-1 NL4-3 Vpr Antiserum	donated by Dr. Jeffrey Kopp.and obtained from the NIH ARP	Cat# 11836
Phospho-Stat5 (Tyr694) (D47E7) XP® Rabbit mAb	Cell Signaling Technologies	Cat# 4322; RRID: AB_10544692
Stat5 (D3N2B) Rabbit mAb	Cell Signaling Technologies	Cat# 25656; RRID: AB_2798908
UNG Mouse Monoclonal Antibody (clone: OTI2C12)	OriGene Technologies	Cat# TA503563; RRID: AB_11126624
VPRBP Polyclonal antibody (DCAF1 antibody)	Proteintech	Cat# 11612-1-AP; RRID: AB_2216933
Anti-Actin antibody	Sigma-Aldrich	Cat# A2066; RRID: AB_476693
Anti-α-Tubulin antibody (clone: DM1A)	Sigma-Aldrich	Cat# T6199; RRID: AB_477583
Alexa Fluor® 488-conjugated AffiniPure F(ab')2 Fragment Donkey Anti-Human IgG (H+L)	Jackson ImmunoResearch	Cat# 709-546-149; RRID: AB_2340569
Alexa Fluor® 488-conjugated AffiniPure F(ab')2 Fragment Donkey Anti-Rabbit IgG (H+L)	Jackson ImmunoResearch	Cat# 711-546-152; RRID: AB_2340619
Alexa Fluor® 488-conjugated AffiniPure F(ab')2 Fragment Goat Anti-Mouse IgG (H+L)	Jackson ImmunoResearch	Cat# 115-546-146; RRID: AB_2338868
Goat anti-Mouse IgG H&L (IRDye® 680RD)	Abcam	Cat# ab216776
Goat anti-Rabbit IgG H&L (IRDye® 800CW)	Abcam	Cat# ab216773
Goat anti-Mouse IgG H&L (IRDye® 800CW)	Abcam	Cat# ab216772
Goat Anti-Rabbit IgG H&L (IRDye® 680RD)	Abcam	Cat# ab216777

**Bacterial and virus strains**		

HIV-1 pNL4.3	donated by Dr M Martin (NIH) and obtained from CFAR	Cat# 2006
HIV-1 pNL4.3 ΔNef	R. Sloan (University of Edinburgh, UK)	Sloan et al., 2011
HIV-1 pNL4.3 ΔVpr	R. Sloan (University of Edinburgh, UK)	Sloan et al., 2011
HIV-1 pNL4.3 ΔVpu	S. Neil (King’s College London, UK)	Neil et al., 2006
HIV-1 pNLENG1-IRES	D. Levy (NYU, USA)	Trinité et al., 2014
HIV-1 pNL4.3 BaL	G. Towers (University College London, UK)	Cat# 100135
HIV-1 pCH040.c/2625	G. Towers (University College London, UK)	Cat# ARP-11740
HIV-1 pCH077.t/2627	G. Towers (University College London, UK)	Cat# ARP-11742
HIV-1 pNL4-3unc-mut4-11	K. Bishop (Francis Crick Institute, UK)	Wanaguru and Bishop, 2021
HIV-1 pNL4-3unc	K. Bishop (Francis Crick Institute, UK)	Wanaguru and Bishop, 2021
HIV-1 pNL4.3 Vpr Q65R	A. Reuschl	This study
HIV-1 pNL4.3 Vpr S79A	A. Reuschl	This study
HIV-1 pNL4.3 Vpr R80A	A. Reuschl	This study

**Biological samples**		

PBMCs isolated from buffy coats from healthy donors	UK NHS Blood and Transplant Service	N/A
Tonsillar tissue from elective tonsillectomy	Imperial College Infectious Diseases Biobank	N/A
Lymph nodes obtained from field surgery of participants undergoing surgery for diagnostic purposes and/or complications of inflammatory lung disease	University of KwaZulu-Natal	N/A
Human Serum from human male AB plasma	Sigma-Aldrich	Cat# H4522-20ML

**Chemicals, peptides, and recombinant proteins**		

Phytohemagglutinin-L (PHA-L)	Sigma	Cat# 11249738001
Interleukin-2 (Human, rDNA derived)	CFAR	Cat# 86/500
Fugene 6 Transfection Reagent	Promega	Cat# E2691
DNase I	Sigma	Cat# DN25-100MG
CellTrace™ Far Red Cell Proliferation Kit	ThermoFisher	Cat# C34564
Fixable Viability Dye eFluor™ 450	ThermoFisher	Cat# 65-0863-14
Recombinant human IL-7	Miltenyi Biotec	Cat# 130-095-362
Recombinant Human IL-15	Peptrotech	Cat# 200-15
Recombinant Human IL-12 p70	Peprotech	Cat# 200-12
Recombinant Human TGF-β1	Peprotech	Cat# 100-21C
T20	CFAR	Cat# 0984
Efavirenz	CFAR	Cat# 0977
Raltegravir	CFAR	Cat# 0980
Ruxolitinib	Selleckchem	Cat# S1378
Zombie NIR™ Fixable Viability Kit	Biolegend	Cat# 423106
Zombie UV™ Fixable Viability Kit	Biolegend	Cat# 423108
Zombie Aqua™ Fixable Viability Kit	Biolegend	Cat# 423102
Super Bright Staining Buffer	ThermoFisher	Cat# SB-4400
Brefeldin A Solution	Biolegend	Cat# 420601
Phorbol 12-myristate 13-acetate (PMA)	Sigma-Aldrich	Cat# P1585-1MG
Ionomycin	Sigma-Aldrich	Cat# I9657-1MG
Intracellular Staining Permeabilization Wash Buffer	Biolegend	Cat# 421002
FOXP3 Fix/Perm Buffer Set	Biolegend	Cat# 421403
True-Phos™ Perm Buffer	Biolegend	Cat# 425401
RLT Buffer (RNeasy Lysis Buffer)	Qiagen	Cat# 79216
β-mercaptoethanol (Sigma-Aldrich)	Sigma-Aldrich	Cat# M3148
Phusion® Hot Start Flex DNA Polymerase	NewEngland Biolabs	Cat# M0535L
TaqMan™ Master-Mix	ThermoFisher	Cat# 4369016
SuperScript™ IV Reverse Transcriptase	ThermoFisher	Cat# 18090050
Fast SYBR™ Green Master Mix	Applied Biosystems	Cat# 4385612
Hoechst33342	ThermoFisher	Cat# H3570

**Critical commercial assays**		

MojoSort™ Human CD4 T Cell Isolation Kit	Biolegend	Cat# 480010
CD45RA MicroBeads, human	Miltenyi Biotec	Cat# 130-045-901

**Deposited data**		

RNAseq data reported in this paper	This study	ArrayExpress: E-MTAB-11454
RNAseq data reported in Kumar et al., 2017	Kumar et al., 2017	GEO: GSE94964

**Experimental models: Cell lines**		

HEK 293 T/17 cells	ATCC	Cat# CRL-11268
Jurkat T cell lines (Clone E6-1)	ATCC	Cat# TIB-152

**Recombinant DNA**		

Flag-tagged NL4.3 Vpr in pcDNA3.1	G. Towers (University College London, UK)	N/A
pWEAU_d15_410_5017	L.E. McCoy (University College London, UK)	N/A
ON-TARGETplus Human DCAF1 siRNA - SMARTpool	Dharmacon	L-021119-01-005
ON-TARGETplus Non-targeting Pool	Dharmacon	D-001810-10-05

**Software and algorithms**		

Image Studio Lite Ver 5.2	Li-Cor	N/A
GraphPad Prism 9	GraphPad	https://www.graphpad.com/
FlowJo v.10.6.2	FlowJo LCC (BD)	https://www.flowjo.com

### Resource availability

#### Lead contact

Further information and requests for resources and reagents should be directed to the lead contact, Professor Clare Jolly (c.jolly@ucl.ac.uk).

#### Materials availability

This study did not generate new unique reagents.

### Experimental model and subject details

#### Cells

Peripheral blood mononuclear cells (PBMC) were isolated from buffy coats from healthy donors (UK NHS Blood and Transplant Service) by density centrifugation using FicollPaque Plus (GE Life Sciences) and cryopreserved in 10% DMSO (Sigma-Aldrich) in 90% FBS (LabTech). Resting CD4+ T cells were isolated from total PBMCs by negative selection using the MojoSort Human CD4^+^ T Cell Isolation kit (Biolegend) according to the manufacturer’s instructions. CD45RA^+^ naïve and CD45RA- memory populations were further separated after CD4^+^ T cell isolation with CD45RA MicroBeads (Biolegend). For activated CD4^+^ T cells, PBMCs were treated with 5 μg/mL PHA (Sigma) and 10 IU/mL IL2 (Centre For AIDS Reagents, National Institute of Biological Standards and Control, UK [CFAR]) in RPMI1640 with 20% FBS for 72 h prior to CD4^+^ T cell isolation. Once purified, CD4^+^ T cells were cultured in RPMI supplemented with 20% FBS and 10 IU/mL IL2. Jurkat T cell lines (Clone E6-1; ATCC TIB-152) were cultured in RPMI with 10% FBS and 100 U/mL penicillin/streptomycin. HEK 293 T/17 cells (ATCC, CRL-11268) were cultured in DMEM with 10% FBS and 100 U/mL penicillin/streptomycin. Tonsil tissue was obtained from an individual with primary HIV infection who underwent routine tonsillectomy (2 months after commencement of ART) or from healthy donors during routine tonsillectomy. As previously described (Thornhill et al., 2019), the tonsillar tissue from elective tonsillectomy was dissected and mechanically digested, prior to cryopreservation of the cellular suspension. This was collected under the Imperial College Infectious Diseases Biobank (REC: 15/SC/0089) and under the GI Illness Biobank Ethics (16/YH/0247). Lymph nodes were obtained from the field of surgery of participants undergoing surgery for diagnostic purposes and/or complications of inflammatory lung disease. Informed consent was obtained from each participant, and the study protocol approved by the University of KwaZulu-Natal Institutional Review Board (approval BE024/09).

### Method details

#### Plasmids, virus and VLP production

The HIV-1 clone pNL4.3 was obtained from the CFAR, NIBSC (cat# 2006). HIV-1 NL4.3 ΔNef and pNL4.3 ΔVpr were provided by R. Sloan (University of Edinburgh, UK) (Sloan et al., 2011). NL4.3 ΔVpu was provided by S. Neil (King’s College London, UK) (Neil et al., 2006). NLENG1-IRES was provided by D. Levy (NYU, USA) (Trinité et al., 2014). NL4.3 bearing the CCR5-tropic BaL *Env* was provided by G. Towers (UCL, UK) (Rasaiyaah et al., 2013). CCR5 tropic transmitter/founder virus plasmids CH044 and CH077 were provided by G. Towers (UCL, UK) and were originally obtained through the NIH AIDS Reagent Program [NIHARP], Division of AIDS, NIAID, NIH: pCH040.c/2625 (cat# 11740) and pCH077.t/2627 (cat# 11742) from Dr. John Kappes and Dr. Christina Ochsenbauer. Plasmids for Vpr packaging mutants pNL4-3unc-mut4-11 (termed HIV-1 Vpr_PM_) and the parental wildtype pNL4-3unc (termed HIV-1 WT_PM_) were provided by K. Bishop (Francis Crick Institute, UK). NL4.3 Vpr Q65R, NL4.3 Vpr S79A, NL4.3 Vpr R80A were generated by site-directed mutagenesis (Promega) using the following primers:

NL4.3 VprQ65R fw:GTGGAAGCCATAATAAGAATTCTGCGACAACTGCTGTTTATCCATTTCAG
NL4.3 VprQ65R rv:CTGAAATGGATAAACAGCAGTTGTCGCAGAATTCTTATTATGGCTTCCAC
NL4.3 Vpr S79A fw: GAATTGGGTGTCGACATGCCAGAATAGGCGTTACTC
NL4.3 Vpr S79A rv:GAGTAACGCCTATTCTGGCATGTCGACACCCAATTC
NL4.3 Vpr R80A fw: GGTGTCGACATAGCGCAATAGGCGTTACTCG
NL4.3 Vpr R80A rv: CGAGTAACGCCTATTGCGCTATGTCGACACC.

All virus and VLP stocks were produced by plasmid transfection of HEK 293 T cells with Fugene 6 (Promega). Supernatants were harvested at 48 h and 72 h, filtered, DNase treated, purified and concentrated by ultracentrifugation through a 25% sucrose cushion and resuspended in RPMI1640 with 10% FBS. Trans-complementation of HIV-1 ΔVpr was performed by co-transfecting 10^6^ HEK 293 T cells with 10 μg pNL4.3 ΔVpr and 2 μg Flag-tagged NL4.3 Vpr in pcDNA3.1 (provided by G. Towers, UCL). For Env-VLP production, 20 μg p8.91 was co-transfected with 10 μg plasmid encoding the HIV-1 T/F envelope pWEAU_d15_410_5017 HIV-1 envelope (provided by LE McCoy, UCL) and 2 μg pcDNA3.1 with or without Flag-tagged NL4.3 Vpr. Viral and VLP titres were determined by measuring reverse transcriptase activity by SG-PERT assay (Pizzato et al., 2009).

#### HIV-1 infection, cell-to-cell spread and Vpr delivery

For cell-to-cell spread experiments, activated primary CD4+ T cells (donor cells) were infected with 800 mU reverse transcriptase per 10^6^ cells of HIV-1 by spinoculation at 1200x*g* for 2 h at room temperature and incubated in RPMI 20% FBS supplemented with 10 IU/mL IL2 for 72 h. HIV-1+ donor CD4^+^ T cells were washed with medium, counted and cultured with autologous primary CD4+ target T cells at a 1:1 ratio in RPMI 20% FBS supplemented with 10 IU/mL IL2 for up to 72 h before analysis by flow cytometry or FACS sorting. Uninfected target CD4^+^ T cells were pre-stained with 1-2 nM CellTrace FarRed dye (Invitrogen) prior to co-culture. For cell-to-cell spread into tonsil-derived lymphocytes, total tonsil lymphocytes were cultured at a 4:1 ratio with HIV-1 infected or uninfected eFluor450-labelled Jurkat T cells. For FACS sorting experiments, donor cells were pre-labeled with cell dye eFluor450 (ThermoFisher). For transwell experiments, HIV-1 infected donor T cells were separated from target T cells by a 0.4 μm transwell insert (Corning). Experiments to quantify cell-to-cell versus cell-free infection in the presence and absence of a transwell were performed in equivalent volumes (600 μL). For some experiments, FACS sorted infected resting CD4^+^ target T cells were returned into cultured for up to 4 days. Infection levels were measured by intracellular Gag staining and flow cytometry, and virus release into cell culture supernatant determined by SG-PERT (Pizzato et al., 2009). At day 1 or day 4 post FACS sorting, resting CD4^+^ T cells were washed extensively and co-cultured at a 1:1 ratio with uninfected eFluor450-labelled Jurkat T cells for 72 h, when Jurkat T cell infection was measured by Gag-staining. Where indicated, cultures were incubated in the presence of 20 ng/mL IL-7 (Miltenyi Biotec), 20 ng/mL IL15 (Peprotech), 20 ng/mL IL12 (Peprotech) or 50 ng/mL TGFβ (Peprotech). The following inhibitors were added 30 min before co-culture at the following concentrations: T20 (25–50 ng/mL, CFAR), Efavirenz (1 μM, CFAR), Raltegravir (5 μM, CFAR) and Ruxolitinib (50 nM, Sigma).

For delivery of Vpr by spinoculation, resting CD4^+^ T cells were incubated with 200–800 mU of virus or Env-VLPs for 15 min at room temperature and subsequently spinoculated at 1200x*g* for 2 h at room temperature. Cells were then cultured as described above.

For RNAi knockdown of DCAF1, primary CD4^+^T cells were activated for 4 days with 1 μg/mL plate-bound αCD3 antibody (cloneOKT3, Biolegend) in the presence of 2 μg/mL soluble αCD28 antibody (clone CD28.2, Biolegend). RNAi knockdown of DCAF1 was performed as described before (Mesner et al., 2020) using ON-TARGET plusHuman DCAF1 siRNA - SMARTpool (Dharmacon) and non-targeting siRNA (Dharmacon) was used as a control. 2 × 10^6^ cells were electroporated with 200 pmol siRNA using a NeonTransfection System (Thermo Fisher Scientific; three pulses, 10 ms, 1600 V). After 48 h, DCAF1 knockdowns were confirmed by western blotting and cells used in cell-to-cell spread experiments as described above.

#### Flow cytometry and FACS

For flow cytometry analysis, cells were washed in PBS and stained with fixable Zombie UV Live/Dead dye, Aqua Live/Dead dye or NIR Live/Dead dye (Biolegend) for 5 min at 37°C. Excess stain was quenched with FBS-complemented RPMI. When tonsil and lymph node lymphocytes were used, Live/Dead staining was quenched using human AB serum (Sigma) in RPMI. Cell surface staining was performed in PBS, complemented with 20% Super Bright Staining Buffer (ThermoFisher) when appropriate, at 4°C for 30 min. Unbound antibody was washed off thoroughly and cells were fixed with 4% FA or PFA before intracellular staining. For intracellular detection of cytokines in infected target CD4^+^ T cells after 72 h of cell-to-cell spread, cells were treated throughout the co-culture with IL-7 and treated with Brefeldin A (Biolegend) for 6 h, if not stated differently, before surface staining and fixation. Where indicated, cells were stimulated with 100 ng/mL PMA (Sigma-Aldrich) and 100 ng/mL Ionomycin (Sigma-Aldrich) for the duration of the Brefeldin A treatment. Permeabilisation for intracellular staining was performed with IC perm buffer or FoxP3 Buffer set (Biolegend) according to the manufacturer’s instructions. For detection of intracellular P-STAT5, cells were resuspended in ice cold True-Phos Perm buffer (Biolegend) and permeabilised for 48 h at −20°C. Intracellular P-STAT5 staining was then performed in PBS with wash steps performed at 1800 rpm for 6 min at 4°C.The following antibody clones and fluorochromes were used: CD3 (UCHT1, Biolegend; BV510, BV711, FITC), CD8 (SK1, Biolegend; BV605, PE), CD4 (SK3, Biolegend; APC/Fire750); CD45RA (HI100, Biolegend; BV421, PE-Dazzle); CD45RO (UCHL1, Biolegend; PerCp-Cy5.5), CD62L (DREG-56, Biolegend, BV785), CD69 (FN50, Biolegend; APC/Fire750, PE-Dazzle); CXCR6/CD186 (K041E5, Biolegend; PE-Dazzle); MCM2 (ab4461, Abcam; was detected with a secondary anti-rabbit AlexaFluor488-tagged antibody); HLA-DR (L243, Biolegend; PerCp-Cy5.5); CD25 (M-A251, Biolegend; PE-Dazzle), CD38 (HIT2, Biolegend, PE-Cy7), CD49a (TS2/7, Biolegend; PE-Cy7); PD-1 (EH12.2H7, Biolegend; PE-Cy7); Ki67 (Ki-67, Biolegend; BV711, PE); Blimp-1 (6D3, BD Pharmingen; PE); CD101 (BB27, Biolegend; PE-Cy7); CX3CR1 (2A9-1, Biolegend; PE-Dazzle); CD103 (Ber-ACT8, Biolegend; Bv711); CD127 (AO19D5, Biolegend; PE-Cy7), IFNγ (B27, Biolegend; PE), Phospho-STAT5 (Clone 47/Stat5 (pY694), BD; PE), Flag-tag (L5, Biolegend; PE) and HIV-1 Gag core antigen (FH190-1-1, Beckman Coulter; PE, FITC). All samples were acquired on either a BD Fortessa X20 or LSR II using BD FACSDiva software and analyzed using FlowJo v10 (Tree Star). Flow cytometry sorting (FACS) was performed with a BD FACSAria III or BD FACSAria IIu Cell Sorter. Cells were either lysed immediately in RLT lysis buffer (Qiagen) with 1% β-mercaptoethanol (Sigma-Aldrich) and stored at −80°C for later RNA extraction or resuspended in RPMI supplemented with 20% FBS and 10 IU/mL IL2 and used immediately.

#### Western blotting

Virus-containing supernatants (normalised for equal loading by measuring RT activity) or 15 μg of total CD4+ T cell protein lysate were separated by SDS-PAGE, transferred onto nitrocellulose and blocked in PBS with 0.05% Tween 20 (v/v) and 5% skimmed milk (w/v). Blots were probed with rabbit antisera raised against HIV-1 Gag p24 (cat# 0432 donated by Dr G. Reid and obtained from the CFAR), Vpr anti-serum (cat# 3951, NIH ARP), α−P-STAT5 (Tyr694) (D47E7, Cell Signaling Technology), α-STAT5 (D3N2B, Cell Signaling Technology), α-beta-Actin (A2066, Sigma-Aldrich), α-UNG (OTI2C12, OriGene Technologies), α−alpha-Tubulin (T6199, Sigma-Aldrich) and α-DCAF1 antibody (11612-1-AO, Proteintech), followed by goat anti-rabbit or goat anti-mouse IRdye 800CW or 680RD infrared secondary antibody (Abcam) and imaged using an Odyssey Infrared Imager (LI-COR Biosciences) and analysed with Image Studio Lite software.

#### Quantification of HIV-1 integration

To quantify integration of HIV-1 in resting T cells, nested Alu-gag quantitative PCR was performed as previously described (Liszewski et al., 2009). Briefly, DNA was isolated from FACS sorted infected resting CD4+ memory T cells after 72 h of cell-to-cell spread using the Qiagen Blood Mini Kit.

Integrated DNA was pre-amplified using 100 nM Alu fw primer, 600 nM HIV-1 Gag rv primer, 0.2 mM dNTP, 1 U Phusion Hot Start Flex (Promega), and 45 ng DNA in 50 μL reactions. Cycling conditions were: 94°C for 30 s, followed by 40 cycles of 94°C for 10 s, 55°C for 30 s, and 70°C for 2.5 min. For quantitation of HIV-1 integration, a second round real-time quantitative PCR was performed using the pre-amplified DNA. These samples were run alongside a standard curve of known dilutions of CEM cells containing integrated HIV-1 DNA. Reactions contained 0.25 μM of RU5 fw and rv primers, and 0.2 μM probes, 1× Qiagen Multiplex Mastermix, and 10 μL pre-amplified DNA. Cyclin conditions were: 95°C for 15 min, followed by 50 cycles of 94°C for 60 s and 60°C for 60 s. 2LTR circles were measured by quantitative PCR (Apolonia et al., 2007). Reactions contained 150 ng DNA, 10μ 2LTR fw and rv primers, 10 μM probe and 1× TaqMan Gene Expression Master Mix (ThermoFisher). Cycling conditions were: 95°C for 15 min, followed by 50 cycles of 95°C for 15 s and 60°C for 90 s. Reactions were performed using 7500 Real-Time PCR System (Applied Biosystems). The following primers and probes were used:

Alu fw: GCCTCCCAAAGTGCTGGGATTACAG
HIV-1 Gag rv: GTTCCTGCTATGTCACTTCC
RU5 fw: TTAAGCCTCAATAAAGCTTGCC
RU5 rv: GTTCGGGCGCCACTGCTAGA
RU5-WT probe: FAM-CCAGAGTCACACAACAGACGGGCACA-TAMRA
RU5-degenerate 1 probe: FAM-CCAGAGTCACATAACAGACGGGCACA-TAMRA
RU5-degenerate 2 probe: FAM-CCAGAGTCACACAACAGATGGGCACA-TAMRA
2LTR fw: AACTAGAGATCCCTCAGACCCTTTT
2LTR rv: CTTGTCTTCGTTGGGAGTGAAT
2LTR probe: FAM-CTAGAGATTTTCCACACTGAC-TAMRA

#### RT-PCR

RNA was extracted from FACS sorted target memory CD4^+^ T cells with RNeasy Micro Kit (Qiagen) according to the manufacturer’s instructions. cDNA was synthesised using SuperScript IV with random hexamer primers (Invitrogen) and qRT-PCR was performed using Fast SYBR Green Master Mix and 7500 Real-Time PCR System (Applied Biosystems). Gene expression was determined using the 2^−ΔΔCt^ method and normalised to GAPDH expression. The following primers were used:

*GAPDH* fw: ACATCGCTCAGACACCATG, rv: TGTAGTTGAGGTCAATGAAGGG;
*CXCR6* fw: GACTATGGGTTCAGCAGTTTCA, rv:GGCTCTGCAACTTATGGTAGAAG;
*PRDM1* fw: ATGCGGATATGACTCTGTGGA, rv: CTGAACCGAAGTACCGCCATC;
*CD69* fw: ATTGTCCAGGCCAATACACATT, rv: CCTCTCTACCTGCGTATCGTTTT;
*S1PR1* fw: TCTGCTGGCAAATTCAAGCGA, rv: GTTGTCCCCTTCGTCTTTCTG;
*KLF2* fw: CTACACCAAGAGTTCGCATCTG; rv: CCGTGTGCTTTCGGTAGTG.

#### Immunofluorescence staining and image analysis

CD4^+^ T cells were spinoculated with HIV-1 WT or ΔVpr virus and incubated in the presence or absence of 1 ng/mL IL-7. At 24 h, cells were adhered onto poly-L-lysine tissue-culture treated CellCarrier Ultra plates (Perkin Elmer) for 1 h and subsequently formaldehyde fixed. For staining, a blocking step was carried out for 1 h at room temperature with 10% goat serum/1% BSA in PBS. STAT5 and P-STAT5 detection was performed by primary incubation with rabbit α-P-STAT5 (Tyr694) (D47E7, Cell Signaling Technology) or rabbit α-STAT5 (D3N2B, Cell Signaling Technology) for 18 h at 4°C and washed thoroughly in PBS. STAT5 or P-STAT5 staining was followed by incubation with mouse α-Gag (HIV-1 Gag core antigen (FH190-1-1, Beckman Coulter) for 18 h at 4°C. Primary antibodies were detected with secondary α-rabbit-AlexaFluor-488 and α-mouse-AlexaFluor-568 conjugates (Jackson Immuno Research) for 1 h at room temperature. All cells were labeled with Hoechst33342 (H3570, Thermo Fisher). Images were acquired using the WiScan® Hermes 7-Colour High-Content Imaging System (IDEA Bio-Medical, Rehovot, Israel) at magnification 60X/1.2NA. Three channel automated acquisition was carried out sequentially. Images were acquired across a well area density resulting in 350–500 FOV/well and 10–20,000 cells. For image analysis, P-STAT5 and STAT5 channels were pre-processed by applying a batch rolling ball background correction in FIJI ImageJ software package (Schindelin et al., 2012) prior to quantification. Cellular intensity of P-STAT5 or STAT5 was quantified using the Athena Image analysis software (IDEA Bio-Medical, Rehovot, Israel). Nuclei were identified as primary objects by segmentation of the Hoechst33342 channel. Cells were identified as secondary objects by nucleus dependent segmentation of the P-STAT-5 or STAT-5 channel. HIV-1 infected Gag^+^ cells were identified by segmenting Gag^+^ signal as primary objects followed by measuring of intracellular Gag intensity. Infected cells were identified by thresholding the population by a minimum Gag^+^ Average intracellular signal of 3.05 × 10^4^ AU/Cell. For all populations, P-STAT-5 and STAT-5 intensity properties were then calculated.

#### Whole transcriptome profiling by RNA-Sequencing

RNA was extracted from FACS sorted target memory CD4^+^ T cells with RNeasy Micro Kit (Qiagen) according to the manufacturer’s instructions. For preparation of RNA-Sequencing libraries, RNA concentration was measured using the Qubit RNA High Sensitivity kit (Life Technologies) and quality checked on the 4200 Tapestation using either the High Sensitivity or standard RNA ScreenTape assay (Agilent Technologies), depending on the measured RNA concentrations. PolyA-tailed mRNA was separated for sequencing during library preparation. Libraries were prepared using KAPA’s mRNA HyperPrep kit (Roche Diagnostics) according to the manufacturer’s instructions using an input of up to 200 ng and a fragmentation incubation time of 8 min at 94°C. Samples were sequenced on Illumina’s NextSeq500 (Illumina Cambridge) using a high output 75 cycle paired-end run. 24 libraries were multiplexed in the same run. Libraries were pooled in equimolar quantities, calculated from concentrations measured using the Qubit dsDNA High Sensitivity kit (Life Technologies) and fragment analysis using the D1000 High Sensitivity assay on the 4200 Tapestation (Agilent Technologies).

RNA sequencing data was quality assessed using FASTQC (https://www.bioinformatics.babraham.ac.uk/projects/fastqc/) before and after low-quality and adapter trimming using Trimmomatic (Bolger et al., 2014). Filtered reads were then pseudo-mapped using Kallisto (Bray et al., 2016) to the transcriptome available in Ensembl v.101 (http://aug2020.archive.ensembl.org/index.html). Per-transcript counts were imported and aggregated per gene using the TXimport R package (Soneson et al., 2016). The DESeq2 package (Anders and Huber, 2010) was used for data normalisation, outlier detection and differential gene expression analysis between biological groups. The DESeq2 results were ranked based on the log_2_ transformation of the adjusted p values, to provide a pre-ranked list for Gene Set Enrichment Analysis (GSEA) (Subramanian et al., 2005) as described in the GSEA documentation. Pathway enrichment and upstream regulator analysis was performed using Gene Set Enrichment Analysis (GSEA) (Subramanian et al., 2005) and Ingenuity Pathway Analysis (IPA) respectively. Heatmaps were generated using ClustVis (https://biit.cs.ut.ee/clustvis/) (Metsalu and Vilo, 2015)

#### Transcriptomic comparison with published human T_RM_ cells

TPM data from previously published transcriptomes of human T_RM_ cells (GSE94964) (Kumar et al., 2017) were summed on gene level with Ensembl gene ID, gene name, and gene biotype using tximport and BioMart (Smedley et al., 2015; Soneson et al., 2016). TPM values < 0.001 were adjusted to 0.001 as a lower limit of detection. These data were aligned to the transcriptomic data from the present study using gene symbol in an integrated log_2_ transformed data matrix and subjected to batch correction by study using Combat (Leek et al., 2012). Expression of selected genes previously identified to be up and downregulated in T_RM_ (Kumar et al., 2017) were used to cluster the samples in both studies using 1-Spearman rank correlation with average linkage in ClustVis (Metsalu and Vilo, 2015). A transcriptional signature score for T_RM_ was derived from the difference between the sum of up and downregulated genes in T_RM_ in the previously published signature. This score was used to evaluate the relative similarity of each transcriptome dataset in the present project to T_RM_ and non-T_RM_ data.

### Quantification and statistical analysis

Statistical analysis was performed using GraphPad Prism. Normally distributed data was analyzed for statistical significance by two-tailed *t-*tests (when comparing two groups) or one-way ANOVA with Bonferroni or Dunnett’s post-test (when comparing more than two groups). Data show the mean +/− the S.E.M with significance shown on the figures. Where appropriate, the median + IQR is shown and Kruskall-Wallis test was used to compare groups. Significance levels were defined as ^∗^, p < 0.05; ^∗∗^, p < 0.01 and ^∗∗∗^, p < 0.001.

## Data Availability

• RNA-Seq data have been deposited at ArrayExpress and are publicly available as of the date of publication. This paper analyses existing, publicly available data. Accession numbers for all datasets are listed in the key resources table. • This paper does not report original code. • Any additional information required to reanalyse data reported in this paper is available from the lead contact upon request.

## Supplemental information

Document S1. Figures S1–S8

Data S1. RNA-seq data of transcriptomes from HIV-1-infected T cells and analysis, related to Figures 5, 6, 7, and S8

Document S3. Article plus supplemental information
