# Supplementary material for: HIV-1 Vpr drives a tissue residency-like phenotype during selective infection of resting memory T cells
Source: Cell Rep. 2022 Apr 13;39(2):110650. doi: 10.1016/j.celrep.2022.110650 (PMC9350556; doi:10.1016/j.celrep.2022.110650)
Supplement: Document S1. Figures S1–S8 [file mmc1.pdf]

Cell Reports, Volume *39*

Supplemental information

HIV-1 Vpr drives a tissue residency-like phenotype during selective infection of resting memory T cells

Ann-Kathrin Reuschl, Dejan Mesner, Maitreyi Shivkumar, Matthew V.X. Whelan, Laura J. Pallett, José Afonso Guerra-Assunção, Rajhmun Madansein, Kaylesh J. Dullabh, Alex Sigal, John P. Thornhill, Carolina Herrera, Sarah Fidler, Mahdad Noursadeghi, Mala K. Maini, and Clare Jolly

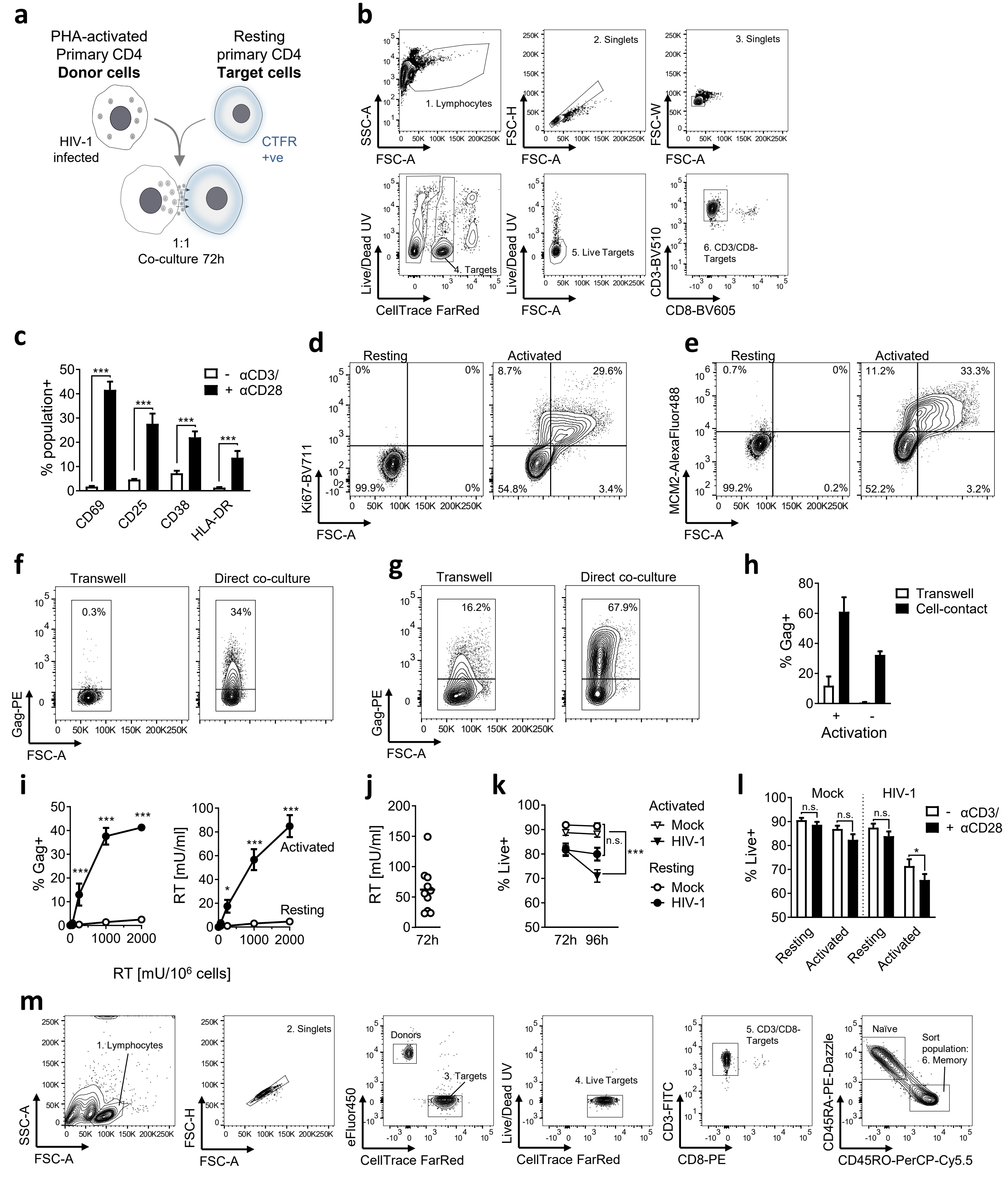

Supplementary data Fig. 1. Related to Figure 1. (**a**) Experimental set-up schematic. (**b**) Flow cytometry gating strategy. (**c**) CD69, CD25, CD38 and HLA-DR expression on resting and activated CD4+ T cells (n=5). (**d**) Ki67 expression on resting and activated primary CD4+ T cells. Representative flow cytometry plots. (**e**) MCM2 expression on resting and activated primary CD4+ T cells. Representative flow cytometry plots. (**f**) Resting or (**g**) mitogenically-activated primary target CD4+ T cells cultured with HIV-1 infected Jurkat T cells separated by a 0.4µm transwell or in direct co-culture. Target cell infection levels was measured by intracellular staining for Gag. Representative flow cytometry plots are shown. (**h**) Infection levels of target CD4+ T cells determined by intracellular Gag staining and flow cytometry (n=2). (**i**) Cell-free infection of resting (open circles) or activated (closed circles) CD4+ T cells with increasing doses of HIV-1 (measured in RT units). Infection levels (%Gag+, left) and virus release (RT units, right) at 72h are shown (n=6). (**j**) Virus release into cultures supernatants during cell-to-cell spread at 72h of co-culture (n=10). (**k**) Survival of resting or activated CD4+ target T cells during cell-to-cell spread over time (n=6). (**l**) Survival or resting and activated CD4+ target T cells during cell-to-cell spread after 24h of αCD3/αCD28 restimulation (n=5). (**m**) Gating strategy for flow cytometry sorting of resting memory target T cells from co-cultures.

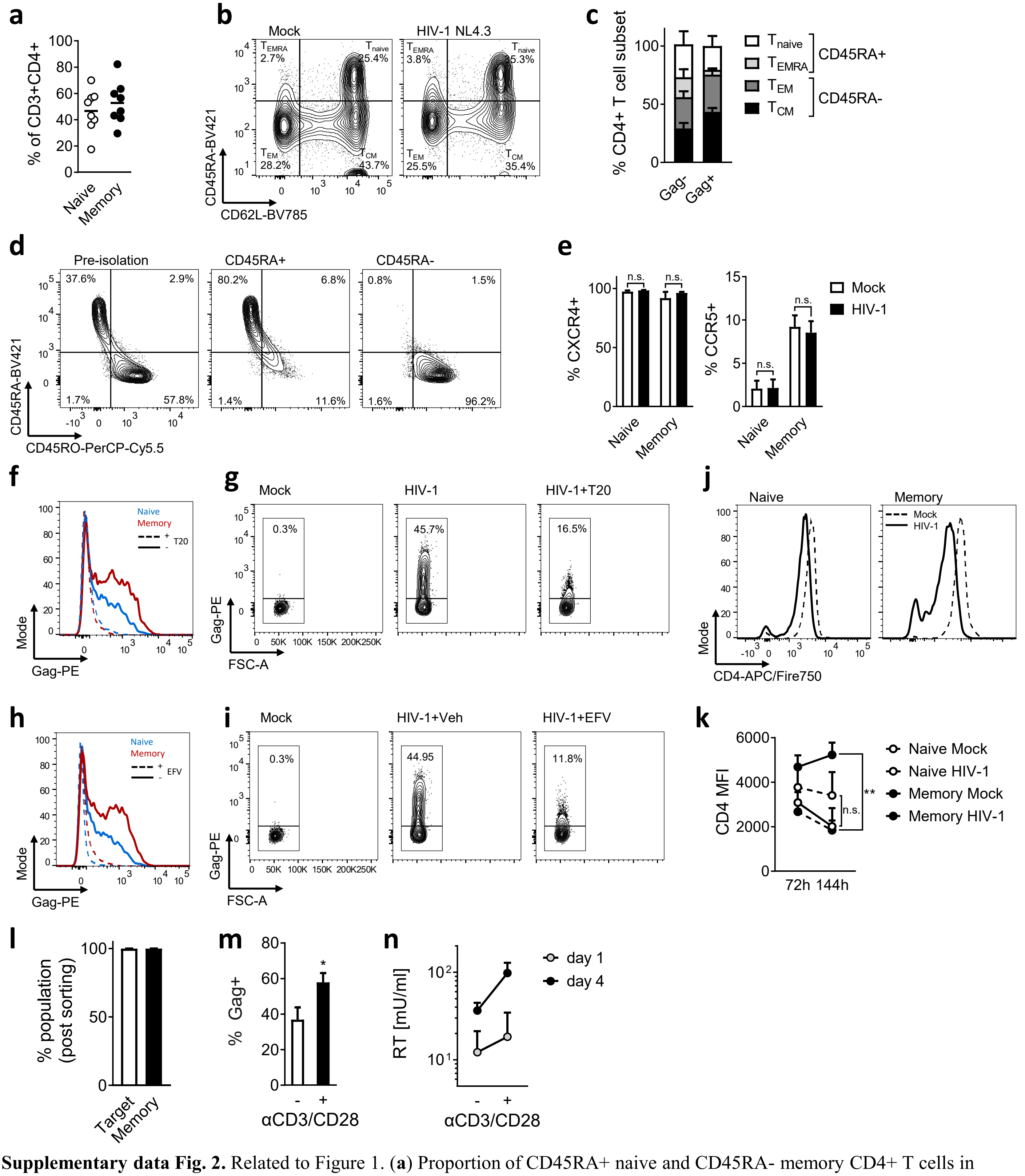

Supplementary data Fig. 2. Related to Figure 1. (**a**) Proportion of CD45RA+ naive and CD45RA- memory CD4+ T cells in unstimulated PBMCs (n=8). (**b**) Resting target CD4+ T cells were cultured with mock-treated or HIV-1-infected donor cells. Surface expression of CD45RA and CD62L were measured after 72h of co-culture. Representative flow cytometry plots are shown. (**c**) Quantification of T cell subsets in infected (Gag+) and uninfected (Gag-) resting CD4+ T cells according to CD45RA/CD62L expression (n=3). (**d**) Representative flow cytometry plots of CD45RA+ and CD45RA- CD4+ T cells pre- and post-isolation. (**e**) Expression of HIV-1 co-receptors CXCR4 (left) and CCR5 (right) receptors on naïve and memory T cells at 72h of co-culture (n=4). (**f**) Representative histogram of intracellular Gag staining in resting naïve (CD45RA+) and memory (CD45RA-) CD4+ T cells after 72h of cell-to-cell spread ± T20. (**g**) Gating strategy for (f). (**h**) Representative histogram of intracellular Gag-levels in resting naïve (CD45RA+) and memory (CD45RA-) CD4+ T cells after 72h of cell-to-cell spread ± Efavirenz. (i) Gating strategy for (h). (**j**) Representative histogram of CD4 surface levels in resting naïve (CD45RA+) and memory (CD45RA-) CD3+ T cells after 72h of cell-to-cell spread. (**k**) HIV-1 infection downregulates CD4 expression. Shown are the CD4 MFI of the total CD3+ target cell population (n=6). (**l**) Mean post-sort population purity of T cells from (Fig. 1 n-p) was 99.92% target cells of which 99.86% were memory T cells (n=5). (**m**) and (**n**) Resting CD4+ memory T cells were isolated after 72h of cell-to-cell spread by FACS sorting and cultured for 1 or 4 days in the presence or absence of αCD3/αCD28. HIV-1 infection was measured by intracellular Gag staining at day 4 (m, n=5) and virus release measured by culture supernatant RT activity (n, n=4).

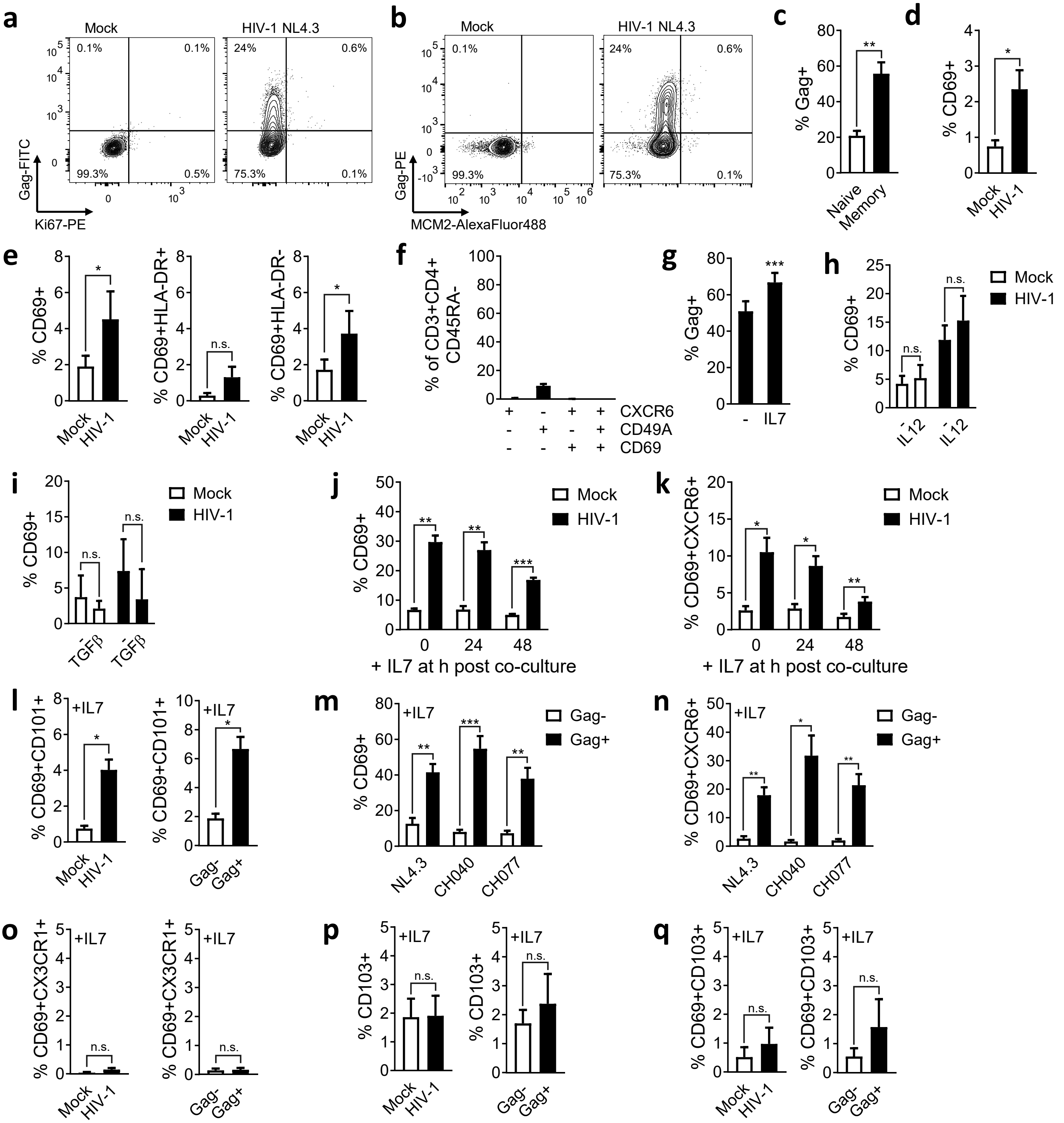

Supplementary data Fig. 3. Related to Figure 2. (**a** and **b**) Representative flow cytometry plots showing (**a**) Ki67 and Gag, or (**b**) MCM2 and Gag staining of resting CD4+ T cells after co-culture with mock or HIV-1 infected primary donor CD4+ T cells. (**c**) FACS sorted CD69- resting naïve or memory CD4+ T cells co-cultured with HIV-1 infected primary CD4+ donor T cells and infection of targets measured by Gag staining (n=4). (**d**) FACS sorted CD69- CD4+ T cells co-cultured with HIV-1 infected primary CD4+ donor T cells. CD69 expression was measured on resting memory CD4+ T cells (n=4). (**e**) Total CD69 expression alongside CD69 with or without HLA-DR co-expression on infected resting memory T cells (n=6). (**f**) Expression of T_{RM}-markers CXCR6, CD49A and CD69 on resting CD4+ memory T cells from unstimulated PBMCs (n=8). (**g**) Quantification of cell-to-cell spread of HIV-1 WT to resting memory CD4+ T cells in the presence or absence of IL7 (n=10). (**h** and **i**) CD69 expression on infected resting memory CD4+ target T cells in the presence or absence of (**h**) IL12 (n=4) or (**i**) TFGβ (n=4). (**j**) CD69 expression on infected resting memory CD4+ T cells with IL7 added at the indicated times post cell-mixing (n=5). (**k**) CD69/CXCR6 co-expression from (**j**) (n=5). (**l**) CD69/CD101 co-expression on infected resting memory CD4+ T cells (n=3) (**m**) CD69 upregulation in response to IL7 on resting memory CD4+ T cells infected HIV-1 NL4.3, or transmitter-founder viruses CH040 and CH077 comparing infected Gag+ and uninfected Gag- bystander cells (n=7). (**n**) CD69/CXCR6 co-expression on resting memory CD4+ T cells from (**i**) (n=7). (**o**) CD69/CX3CR1 co-expression on infected resting memory CD4+ T cells (n=3). (**p**) CD103 expression and (**q**) CD69/CD103 co-expression on infected resting memory CD4+ T cells (n=4). Data are the mean±SEM. Paired two-tailed *t*-test or one-way ANOVA with Bonferroni post-test was used. For (**i**), median+IQR is shown and Kruskall-Wallis test was used to compare groups *, $p<0.05$; **, $p<0.01$; ***, $p<0.001$; n.s., not significant.

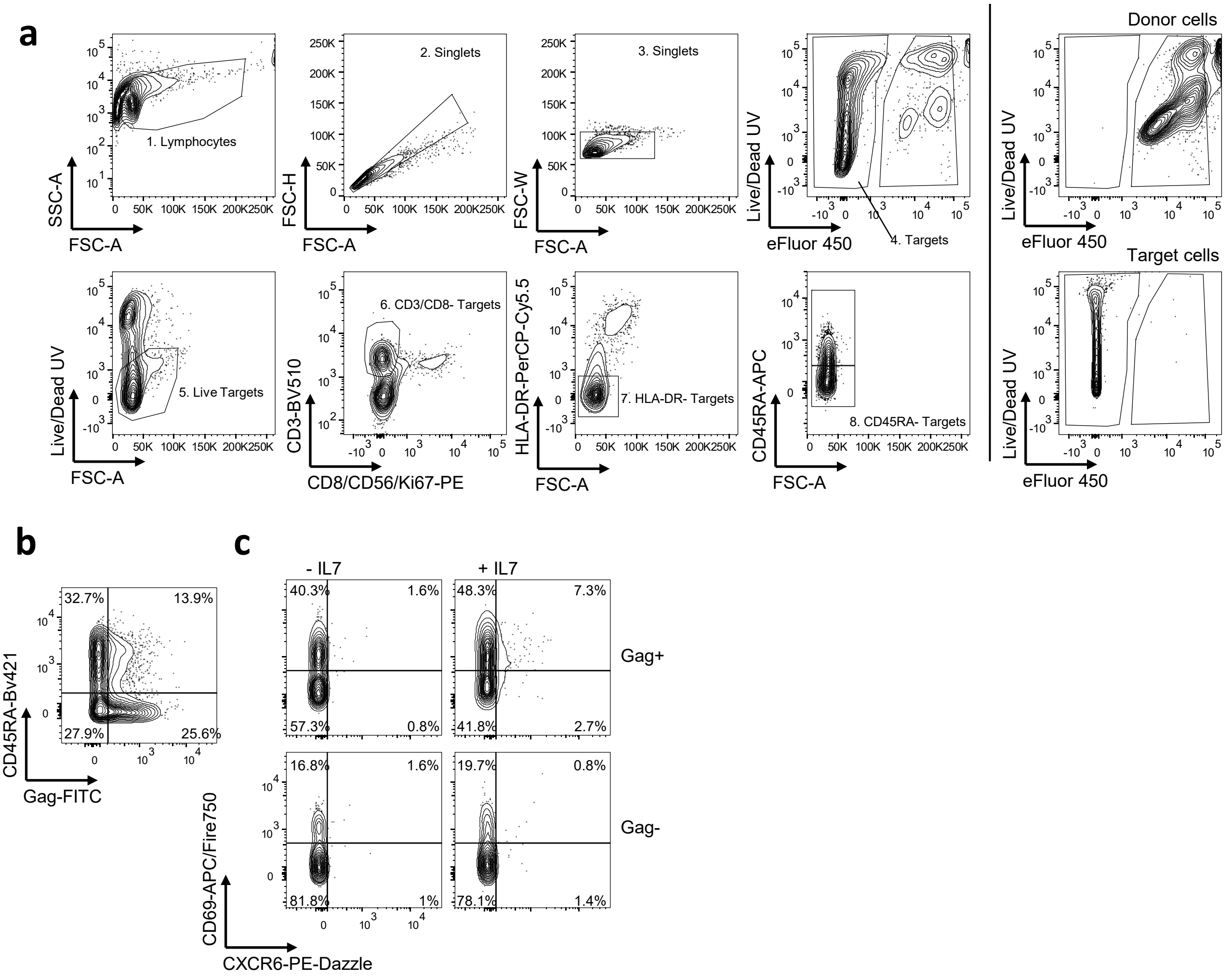

Supplementary data Fig. 4. Related to Figure 2. (**a**) Flow cytometry gating strategy for infection of tonsil-derived T cells by cell-to-cell spread. (**b**) Total lymphocytes from one mediastinal lymph node sample co-cultured with HIV-1 infected activated autologous LN-derived lymphocytes. Targets gated as in (a). Infection of resting CD4+ T cells (CD3+/CD8-/Ki67- lymphocytes) shown as CD45RA vs Gag. (**c**) CD69 and CXCR6 co-expression on infected Gag+ and uninfected Gag- lymph node resting memory CD4+ T cells ±IL7.

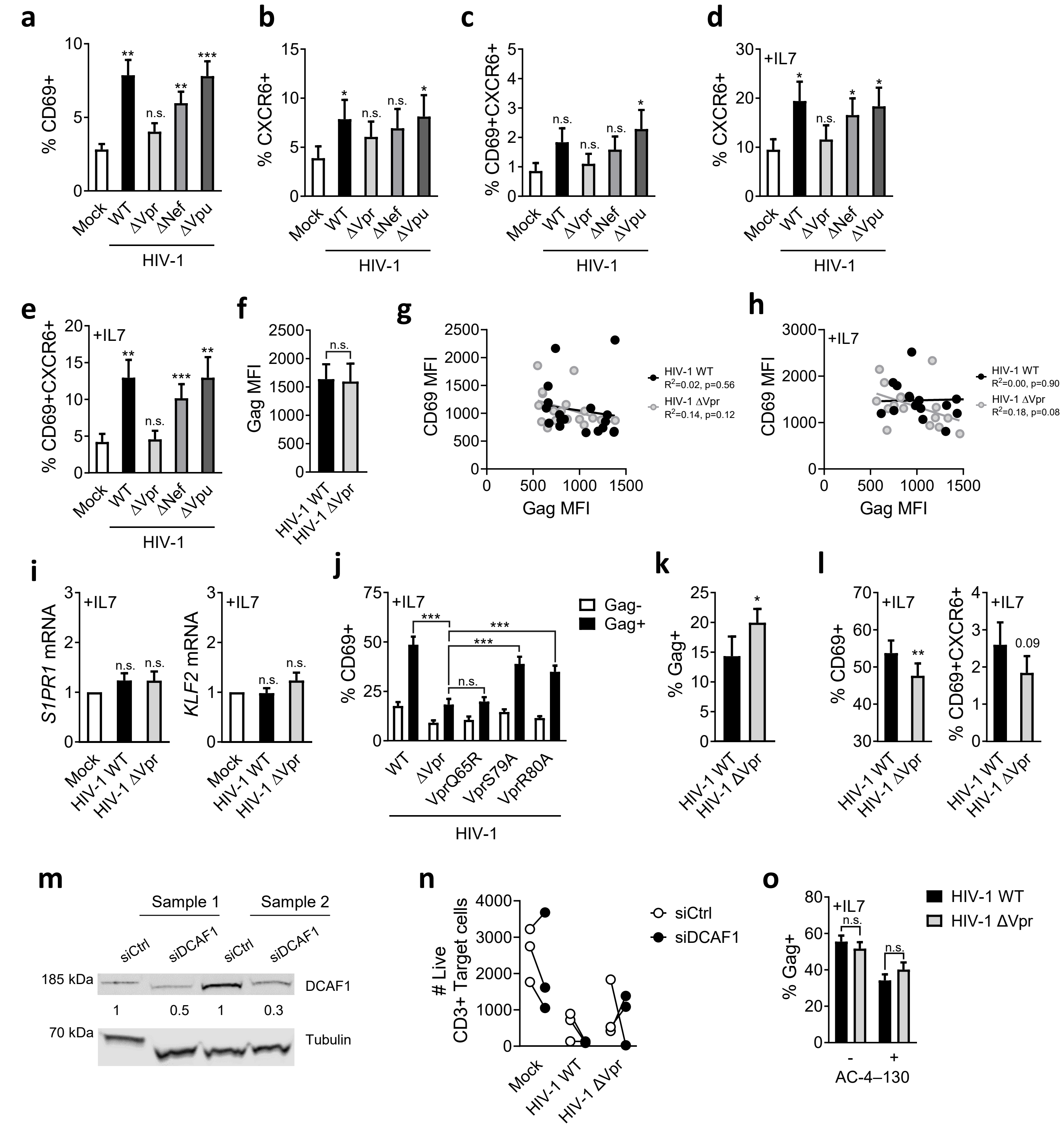

Supplementary data Fig. 5. Related to Figure 3 and 7. (**a**) CD69 surface expression on resting CD45RA- memory CD4+ T cells following co-culture with primary CD4+ donor T cells infected with HIV-1 NL4.3 (WT), ΔVpr, ΔNef or ΔVpu or uninfected (mock) donors (n=9). (**b**) CXCR6 expression from (**a**) (n=9). (**c**) CD69/CXCR6 co-expression from (**a**) (n=9). (**d**) As for (b) but cells were incubated in the presence of IL7 (n=9). (**e**) CD69/CXCR6 surface co-expression from (d) (n=9). (**f**) Gag MFI of cell-to-cell spread of HIV-1 WT and ΔVpr to resting memory CD4+ T cells (n=10). Correlation plot of CD69 MFI with Gag MFI in (**g**) or presence (**h**) of IL7 (n=18). (**i**) *S1PR1* and *KLF2* mRNA levels in FACS sorted resting memory CD4+ T cells from Fig. 3e. Fold change over mock is shown (n=5). (**j**) CD69 upregulation in response to IL7 on resting memory CD4+ T cells infected with HIV-1 WT, ΔVpr or Vpr mutants, categorised by Gag expression (n=9). (**k**) Infection levels of resting memory T lymphocytes from cellularised tonsils co-cultured with HIV-1 WT or ΔVpr infected Jurkat T cells (l) CD69 (left) and CD69/CXCR6 (right) expression on cells from (k) in response to IL7 (n=4). (**m**) Western blot showing siRNA knockdown of DCAF1 in CD3/CD28-activated CD4+ T cells 48h post transfection. Two representative samples are shown. (**n**) Number of live CD3+ Target T cells recovered after 72h of cell-to-cell spread into control or DCAF1 siRNA-treated T cells (n=3). (**o**) Quantification of cell-to-cell spread of HIV-1 WT and ΔVpr to resting memory CD4+ T cells in the presence IL7 ± AC-4-130 (n=6).

a

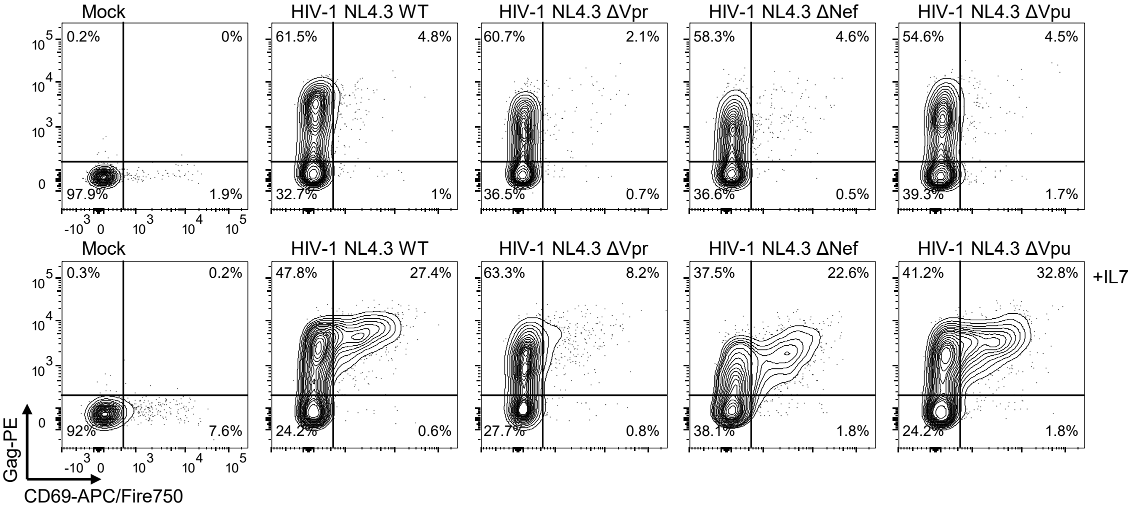

b

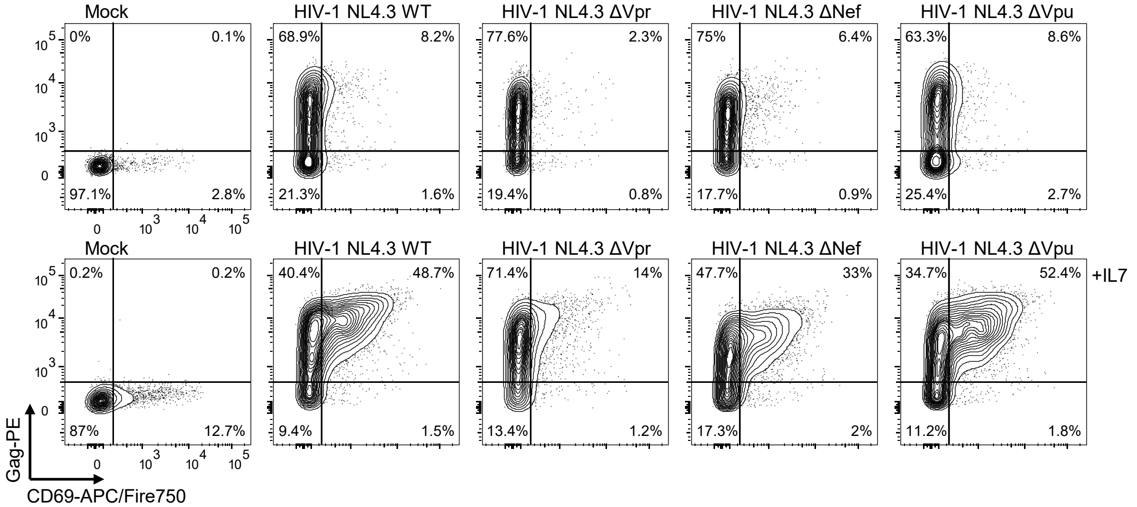

c

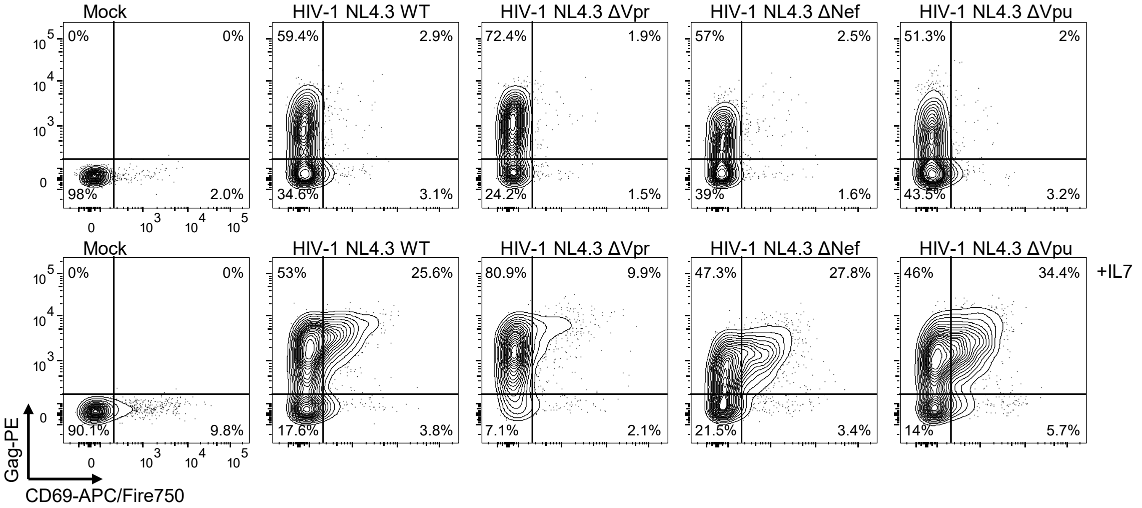

Supplementary data Fig. 6. Related to Figure 3. Resting memory CD4+ T cells were co-cultured with HIV-1 infected primary CD4+ T cells infected with HIV-1 WT or mutant viruses. Representative flow cytometry plots of HIV-1 Gag and CD69 co-expression in the presence or absence of IL7 from three independent experiments are shown (**a-c**).

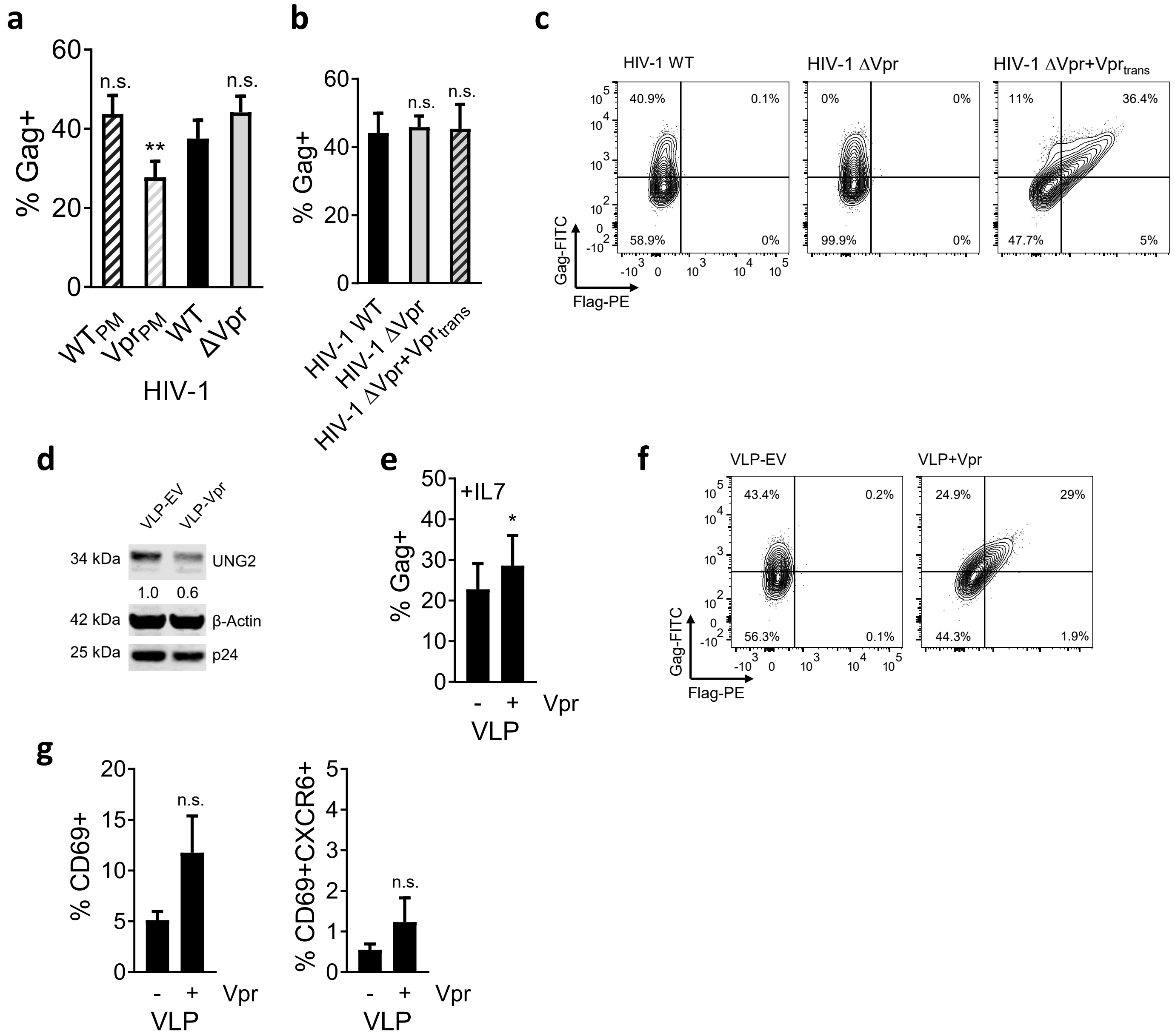

Supplementary data Fig. 7. Related to Figure 4 and 7. (**a**) Quantification of cell-to-cell spread of the indicated viruses to resting memory CD4+ T cells at 72h of co-culture (n=8). (**b**) Quantification of intracellular Gag-levels in resting memory CD4+ T cells at 72h post spinoculation with the indicated viruses (n=10). (**c**) Representative flow cytometry plots for (b) of virus delivery by spinoculation. (**d**) Western blot showing the degradation of Vpr-target UNG2 in Jurkat T cells 24h post spinoculation with Env-VLPs±Vpr. (**e**) Quantification of intracellular Gag-levels in resting memory CD4+ T cells at 72h post spinoculation with VLPs ± Vpr (n=5). (**f**) Representative flow cytometry plots for (e) of VLP-delivery of flag-tagged Vpr by spinoculation. (**g**) Expression of CD69 (left) and CD69/CXCR6 (right) on Gag+ resting memory CD4+ T cells at 72h post spinoculation of Env-VLPs with or without Vpr in the absence of IL7 (n=5).

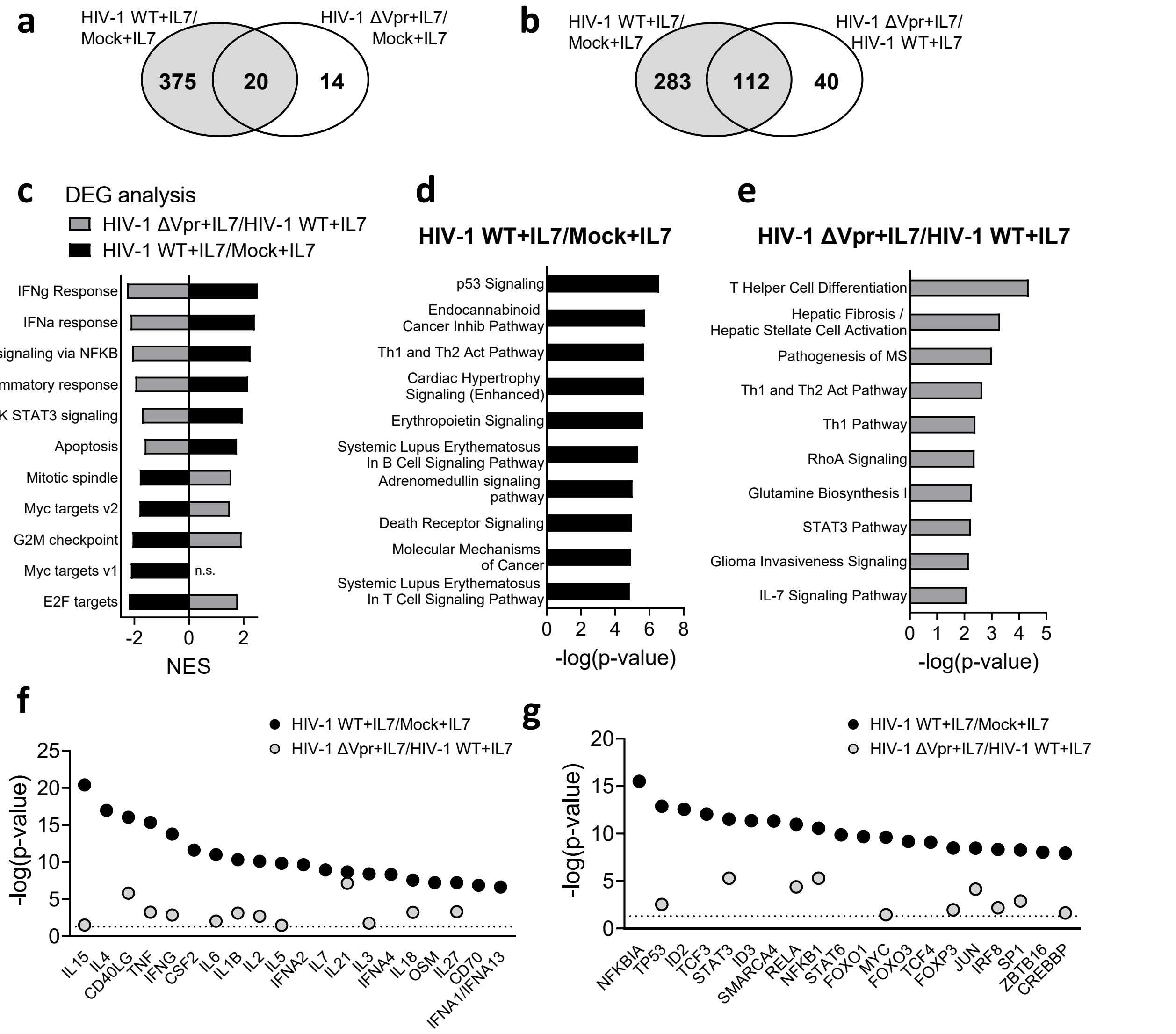

Supplementary data Fig. 8. Related to Figure 5. (**a**) and (**b**) Venn diagrams showing overlap of DEGs comparing expression profiles of HIV-1 WT+IL7/Mock+IL7 with (a) HIV-1 ΔVpr+IL7/Mock+IL7 or (b) HIV-1 ΔVpr+IL7/HIV-1 WT+IL7. (**c**) GSEA was performed on expression profiles comparing HIV-1 WT+IL7/Mock+IL7 (black) or HIV-1 ΔVpr+IL7/HIV-1 WT+IL7 (grey). Normalised enrichment scores are shown for significantly enriched Hallmark gene sets are shown (FDR q-value<0.05 and NES>1.75). (**d**) and (**e**) top ten significantly enriched canonical pathways predicted by ingenuity pathway (IPA) analysis of DEGs (d) HIV-1 WT+IL7/Mock+IL7 or (e) HIV-1 ΔVpr+IL7/HIV-1 WT+IL7 (adjusted p-value<0.05). (**f**) Cytokines and (**g**) transcription regulators predicted to be upstream regulators by IPA of gene expression signatures HIV-1 WT+IL7/Mock+IL7 (black) or HIV-1 ΔVpr+IL7/Mock+IL7 (grey), line indicates p=0.05.
